# Male meiotic spindle poles are stabilized by TACC3 and cKAP5/chTOG differently from female meiotic or somatic mitotic spindles in mice

**DOI:** 10.1038/s41598-024-55376-z

**Published:** 2024-02-27

**Authors:** Calvin Simerly, Emily Robertson, Caleb Harrison, Sydney Ward, Charlize George, Jasmine Deleon, Carrie Hartnett, Gerald Schatten

**Affiliations:** Departments of Cell Biology, Ob-Gyn-Repro Sci, and Bioengineering, Pittsburgh Development Center of Magee-Womens Research Institute, University of Pittsburgh Medical Center, 204 Craft Avenue, Pittsburgh, PA 15213 USA

**Keywords:** Cell biology, Cytoskeleton

## Abstract

Transforming acidic acid coiled-coil protein 3 (TACC3) and cytoskeleton associated protein 5 (cKAP5; or colonic hepatic tumor overexpressed gene, chTOG) are vital for spindle assembly and stabilization initiated through TACC3 Aurora-A kinase interaction. Here, TACC3 and cKAP5/chTOG localization with monospecific antibodies is investigated in eGFP-centrin-2- expressing mouse meiotic spermatocytes. Both proteins bind spermatocyte spindle poles but neither kinetochore nor interpolar microtubules, unlike in mitotic mouse fibroblasts or female meiotic oocyte spindles. Spermatocytes do not display a liquid-like spindle domain (LISD), although fusing them into maturing oocytes generates LISD-like TACC3 condensates around sperm chromatin but sparse microtubule assembly. Microtubule inhibitors do not reduce TACC3 and cKAP5/chTOG spindle pole binding. MLN 8237 Aurora-A kinase inhibitor removes TACC3, not cKAP5/chTOG, disrupting spindle organization, chromosome alignment, and impacting spindle pole γ-tubulin intensity. The LISD disruptor 1,6-hexanediol abolished TACC3 in spermatocytes, impacting spindle bipolarity and chromosome organization. Cold microtubule disassembly and rescue experiments in the presence of 1,6-hexanediol reinforce the concept that spermatocyte TACC3 spindle pole presence is not required for spindle pole microtubule assembly. Collectively, meiotic spermatocytes without a LISD localize TACC3 and cKAP5/chTOG exclusively at spindle poles to support meiotic spindle pole stabilization during male meiosis, different from either female meiosis or mitosis.

## Introduction

Understanding the mechanism of spindle assembly and maintenance between male or female meiosis and mitosis, including commonalities and distinctions, will permit a greater understanding of how we can avoid chromosomal separation errors that lead to infertility, genetic and developmental defects, miscarriages, and cancers. Such studies remain exciting avenues for understanding the fundamental differences in meiotic spindle assembly and stabilization between male and female meiosis and somatic cell mitosis. Accurate bipolar spindle assembly in meiosis and mitosis is vital for faithful chromosome alignment and segregation to avoid generating chromosomal aneuploidy errors that can form compromised cells or gametes.

Tightly choregraphed, precise functioning and assembly/disassembly of the spindle for correct chromosome segregation differs between classical mammalian mitosis and female or male meiosis^1–4^. Mammalian mitotic cells largely utilize centrosomes, the cells major microtubule organizing centers (MTOC’s), composed of a pair of centrioles surrounded by pericentriolar material (PCM) to nucleate spindle microtubules for assembling the bipolar spindle^5–7^. By contrast, meiotic spindles in oocytes of the most studied mammal, the mouse, utilize maternally derived spindle pole microtubule organizing centers (MTOCs) lacking canonical centrioles to direct meiotic spindle assembly^2,8–12^. Mouse meiotic oocytes lose their functional maternal centriole replication activity prior to meiosis resumption^13,14^, although recent studies have found maternal centriole remnants at mouse meiotic spindle poles raising conundrums regarding what roles, if any, maternal centrioles may play in female meiosis^15^. In mouse male meiosis, spermatocytes have canonical centrosomes at their spindle poles that play significant roles in meiotic spindle assembly and maintenance^16–18^. But mouse spermatocytes also undergo unusual centriole events, with two rounds of centriole duplication occurred in meiosis- I spermatocytes as opposed to a single centriole event per cell cycle observed in mitosis and maturation, where the acquisition of distal proteins that mark full centriole maturation was found on all four derived centrioles at metaphase-I rather than taking one-and-a-half cell cycles to acquire these structures as observed in mitotic cells^18–21^. Thus, male meiosis differs fundamentally from both female meiosis and mitosis and is not well understood.

Mitotic spindle microtubule assembly and stabilization as well as centrosome spindle pole integrity is supported by numerous important accessory proteins, although the mechanistic understanding of their roles is not well understood^6^. Transforming acidic coiled-coil containing protein 3 (TACC3) is a centrosomal protein involved in microtubule nucleation and stabilization of gamma-tubulin ring complex (γ-TuRC) proteins in many vertebrate cells^22–28^. TACC3 frequent binds in a complex with cKAP5/chTOG, a family member of *Xenopus* microtubule-associated protein 215 (XMAP215) that acts as a microtubule polymerase to regulate mitotic spindle microtubule dynamics^29–32^. TACC3 is regulated through phosphorylation of a conserved amino acid site by Aurora A kinase (AURKA) to conduct the self-assembled TACC3-cKAP5/chTOG complex to centrosomes or to intact spindle microtubules where the dyad can form a tricomplex with clathrin to crosslink and stabilize adjacent kinetochore microtubules to stabilize chromosome interactions^23,33–37^. Additionally, the TACC3-cKAP5/chTOG dyad may also function as a plus-end tracking protein to regulate mitotic spindle dynamics in somatic cells^38^. But TACC3 and cKAP5/chTOG can also act independently at the plus-ends of spindle microtubules in mitotic somatic cells to increase the lateral attachment of KTMTs on chromosomes for directing chromosome congression and biorientation on spindles or to correct microtubule errors at the kinetochores to ensure accurate mitotic chromosome segregation^39,40^.

In meiotic oocytes, TACC3 and cKAP5/chTOG proteins directly bind female meiotic spindle pole microtubules to support spindle stabilization during chromosomal reductional divisions^41–46^. Recently, careful studies in meiotic oocytes have identified a unique membrane-less LISD encompassing most mammalian meiotic oocyte spindles that harbors 19 critical microtubule regulatory proteins important for bipolar spindle assembly and maintenance in an entity that surrounds the meiotic spindle region nestled within the very large oocyte volume. This permits the quick shuttling of vital spindle support proteins to the spindle lattice during meiosis^47^. Among the essential constituents necessary for LISD assembly in mammalian oocytes, shown by pharmacological agents and endogenous protein disruption experiments, are TACC3, AURKA, and clathrin. Additionally, cKAP5/chTOG has also been identified in the LISD^47^.

Oocytes experiments from kinase knockout mutant mice lacking polo-like kinase 1 (plk-1) or AURKA demonstrate abnormal first meiotic bipolar spindle assembly and chromosomal alignment errors, partially related to the inability to assembly the LISD^44,45^. Additionally, a transgenic mouse engineered without spindle pole MTOCs and lacking the ability to concentrate AURKA at their poles could not assemble a LISD, causing altered spindle structures with misaligned chromosomes^46^. Overall, TACC3 in mammalian meiotic oocytes is required for efficient microtubule assembly, especially kinetochore and intrapolar spindle microtubule functions during bivalent chromosome reductional divisions, a crucial time when meiotic chromosomal errors can occur^2,48^.

In mouse meiotic spermatocytes, TACC3 was reported at spindle pole centrioles under the control of the cell cycle kinase Aurora A^18^. Using conditional mouse knockout strategies, loss of polo-like kinase 1 (PLK1) and AURKA in mouse spermatogenic cells caused depleted spindle pole TACC3, male meiotic dysfunctional spindle pole separation, and aberrant centriolar and γ-tubulin recruitment to active centrosomes. Thus, these cell cycle kinases are vital to centrosome replication, maturation, and function for directing accurate meiotic spindle assembly in mouse spermatogenesis.

Here, we provide additional evidence of the role of TACC3 and its frequent binding partner cKAP5/chTOG in male meiotic spermatocytes from mice expressing GFP centrin-2 protein (GFP CETN2) in centrioles to demark their spindle poles. We utilize highly specific antibodies to trace TACC3 and cKAP5/chTOG in mouse meiotic spindles, showing restricted localization of these proteins to the spindle pole centrosomes that differ from localization reports in mouse female meiotic spindles or mitotic fibroblasts. We probe how TACC3 spindle pole disruption with AURKA inhibitors, the liquid condensate disruptor 1,6 hexanediol, and microtubule inhibitors impacts male meiotic spindle integrity. We also investigate TACC3 recruitment in mouse spermatids fused into mouse female oocytes. Collectively, our results suggest a more restricted distribution of TACC3 and cKAP5/chTOG in male meiotic spindles compared to female meiotic or somatic mitotic cells with a more prominent role for this dyad in spindle pole organization and stabilization post-assembly than for initial centrosome spindle microtubule generation. These findings may provide important spindle mechanistic insights for avoiding production of aberrant male gametes that have severe consequences for aneuploidy, developmental defects, abortion, and male infertility.

## Results

Two monospecific TACC3 and one polyclonal rabbit cKAP5/chTOG antibody was used to track these spindle proteins in mouse spermatogenic cells (Suppl Fig. 1S). We relied primarily on the rabbit monoclonal TACC3 antibody, owing to its greater specificity in immunolabeling experiments using somatic mouse CF-1 fibroblasts and meiotic oocytes in mice. The distinctions in the staining patterns observed in these different cell types investigated along with antibody validation criteria (Suppl Fig. S1) provided confidence in these antibodies to track TACC3 and cKAP5/chTOG in spermatocytes.

**Figure 1:**
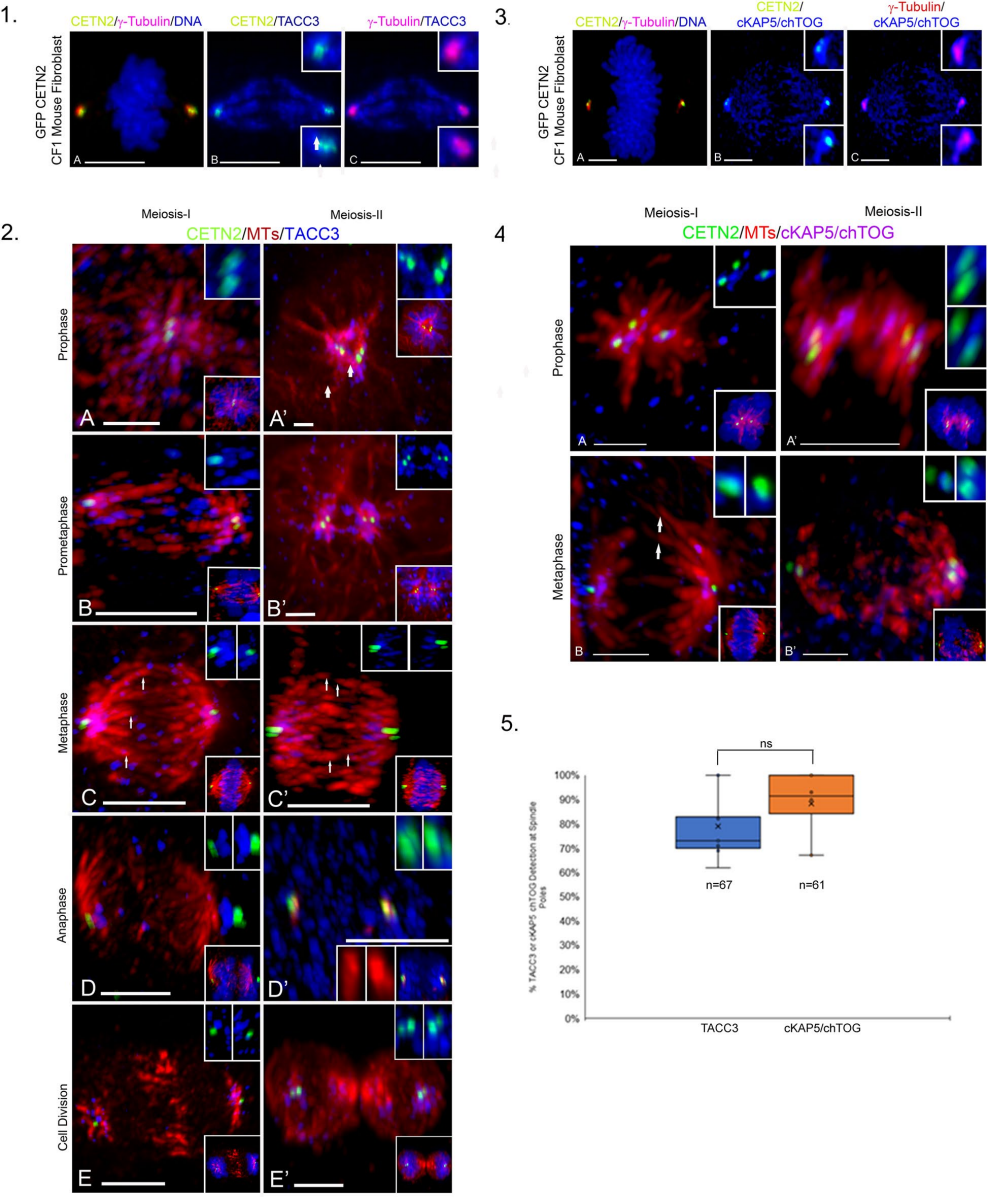
Unlike Mouse CF-1 Fibroblast Cells, TACC3 and cKAP5/chTOG immunolabel only mouse spermatogenic meiotic spindle poles but do not immunostain pre-prophase, interkinesis or post-meiotic spermatids including testicular sperm*.* *Panel 1*: Metaphase GFP CETN2 CF-1 mouse fibroblast (**A**, **B**: green) immunolabeled with γ-tubulin (**A**, **C**: red), anti-TACC3 (**B**, **C**: blue) and DNA (**A**: inset, blue). TACC3 immunolabels the entire spindle including spindle kinetochore microtubules (**B**, **C**, arrows). Insets: details; spindle pole TACC3 (blue), GFP-CETN2 centrioles (**B**; green) and γ-tubulin (**C**: red). *Panel 2*: Meiosis I (**A**–**E**) and meiosis-II (**A’**–**E’**) GFP CETN2-expressing spermatocytes (green), microtubules (red) and TACC3 (blue). TACC3 concentrates at metaphase spindle poles (**C**, **C’**: blue) but not kinetochore microtubules (**C**–**C’**: red, arrows). Upper insets: spindle pole TACC3 (blue) and GFP CETN2 centrioles (green); lower insets: GFP CETN2 (green), microtubules (red) and DNA (blue), except **D’**: left, middle insets: microtubules, red; right inset: GFP CETN2 centrioles (green) and DNA (blue). *Panel 3*: Metaphase GFP CETN2 CF-1 mouse fibroblast (**A**, **B**: green) immunolabeled with γ-tubulin (**A**, **C**: red), anti-cKAP5/chTOG (**B**, **C**: blue) and DNA (**A**: inset; blue). cKAP5/chTOG immunolabels the entire spindle including kinetochore microtubules (**B**, **C**, arrows). Insets (**B**, **C**): details, spindle pole cKAP5/chTOG (blue), centrioles (**B**: green) and γ-tubulin (**C**: red). *Panel 4:* Meiosis-I (**A**, **B**) and meiosis-II (**A’**–**B’**) GFP CETN2-expressing spermatocytes (green), microtubules (red) and cKAP5/chTOG (blue). cKAP5/chTOG immunolabels spindle poles, not spindle kinetochore microtubules (**B**: arrows). Upper insets: spindle pole GFP CETN2 centrioles (green) and cKAP5/chTOG (blue); lower insets: GFP CETN2 (green), microtubules (red) and DNA (blue). *Panel 5*: Graph, right: analysis of > 60 TACC3 or cKAP5/chTOG meiotic spermatocytes showing similar TACC3 or cKAP5/chTOG spindle pole localization (ns, not significant; *p* < 0.5). All scale bars = 5 µm.

In GFP CETN2-expressing fibroblast cells at metaphase, TACC3 and cKAP5/chTOG strongly circumscribed centrosomes/ spindle poles and immunolabeled kinetochore and intrapolar microtubules (Fig. 1, panel 1 A–C and panel 3 A–C), as previously reported in other somatic cells^28^. TACC3 and cKAP5/chTOG did not immunolabel microtubule plus ends near kinetochores, unlike other reports^38–40^. Conversely, in GFP CETN2-expressing mouse spermatocytes at either meiosis-I or meiosis-II, TACC3 and cKAP5/chTOG first coalesced around monastral microtubules at prophase before concentrating at the metaphase spindle poles without immunolabeling spindle microtubules, including kinetochore microtubules (KTMT’s), or the plus ends of microtubules near the kinetochores (Fig. 1, panels 2 and 4). We detected no LISD domain in spermatocyte meiotic spindles, as described in female meiotic oocytes^47^. Analysis showed that > 85% of male meiotic cells co-expressed spindle pole TACC3 and cKAP5/chTOG (Fig. 1, panel 5).

Several spermatocyte stages lacked TACC3 immunolabeling (Suppl Fig. S2). In prophase-I spermatocytes prior to nuclear envelop breakdown (NEBD), the synaptonemal complex antibody SYCP3 marked prophase-I substages but without TACC3 detection, including in post-leptonema cells after first centriole duplication (Suppl Fig. S2, panel 1; insets)^18^. During interkinesis between meiosis- I and- II, when the second round of centriole duplication is completed^18^, TACC3 was not identified at the centrosomes or interphase microtubules (Suppl Fig. S2, panel 2). Finally, TACC3 was not observed in post-meiotic round spermatids, elongating spermatid with manchette microtubules, or in testicular spermatozoa (Suppl Fig. S2, panel 3).

Distinct from male meiosis, female meiosis displays extensive TACC3 and cKAP5/chTOG staining profiles represented here in GFP CETN2- expressing mice (Suppl Fig. S3). TACC3 is absent in mature GV-arrested oocytes and attached somatic cumulus cells that support oocyte maturation (Suppl Fig. S3A–C, arrows). Just prior to meiosis resumption, when pericentrin-labeled MTOC’s start expansion along the GV-nuclear surface, TACC3 strongly labels both GV-bound and cytoplasmic MTOC’s, including those expressing GFP CETN2 that often nucleate small astral microtubules^15^ (Suppl Fig. S3D–F). Non-mitotic somatic cumulus cells, however, never express TACC3 (Suppl Fig. S3D–F and G–H). In early prometaphase-I, a large TACC3 LISD engulfs the female meiotic spindle as the bivalents condense (Suppl Fig. S3G) and the fragmented MTOC’s segregate to the developing spindle poles, one containing maternal GFP CETN2-expressing foci (Suppl Fig. S3H,I). TACC3 encircles, but does not directly co-immunolabel, the cytoplasmic MTOCs after meiosis resumption (Suppl Fig. S3G–I). By late prometaphase-I, TACC3 concentrates in the LISD and spindle pole microtubules as the fragmented spindle-associated MTOCs with retained maternal GFP CETN2-expressing foci coalesce at both poles (Suppl Fig. S3J–L). The spindle pole MTOC’s and maternal GFP CETN2-expressing foci do not immunolabel directly with TACC3^46^. By metaphase-II arrest, TACC3 labels all spindle microtubules, including KTMTs (Suppl Fig. S3N,O), though not spindle pole MTOCs with retained maternal GFP CETN2-expressing foci (Suppl Fig. S3M–O). The cytoplasmic asters in the metaphase-II oocyte (cytasters) lose circumscribing TACC3 foci observed earlier in first meiosis (Suppl Fig. S3M–O).

We also traced cKAP5/chTOG during female meiosis (Suppl Fig. S4). cKAP5/chTOG bound the mature GV nuclear surface and nuclear envelop invagination sites, but not the GV-bound MTOC’s or cumulus cells (Suppl Fig. S4B,C). By late prometaphase I, cKAP5/chTOG strongly labels the LISD and spindle microtubules, but not the spindle pole MTOCs (Suppl Fig. S4D–F). The largest cortical cytoplasmic MTOCs also co-immunolabeled with cKAP5/chTOG, unlike TACC3 (Suppl Fig. S4D–F). These results confirm and extend previous reports on TACC3 and cKAP5/chTOG localization in mouse meiotic oocytes^41,43–47,49,50^.

**Figure 2:**
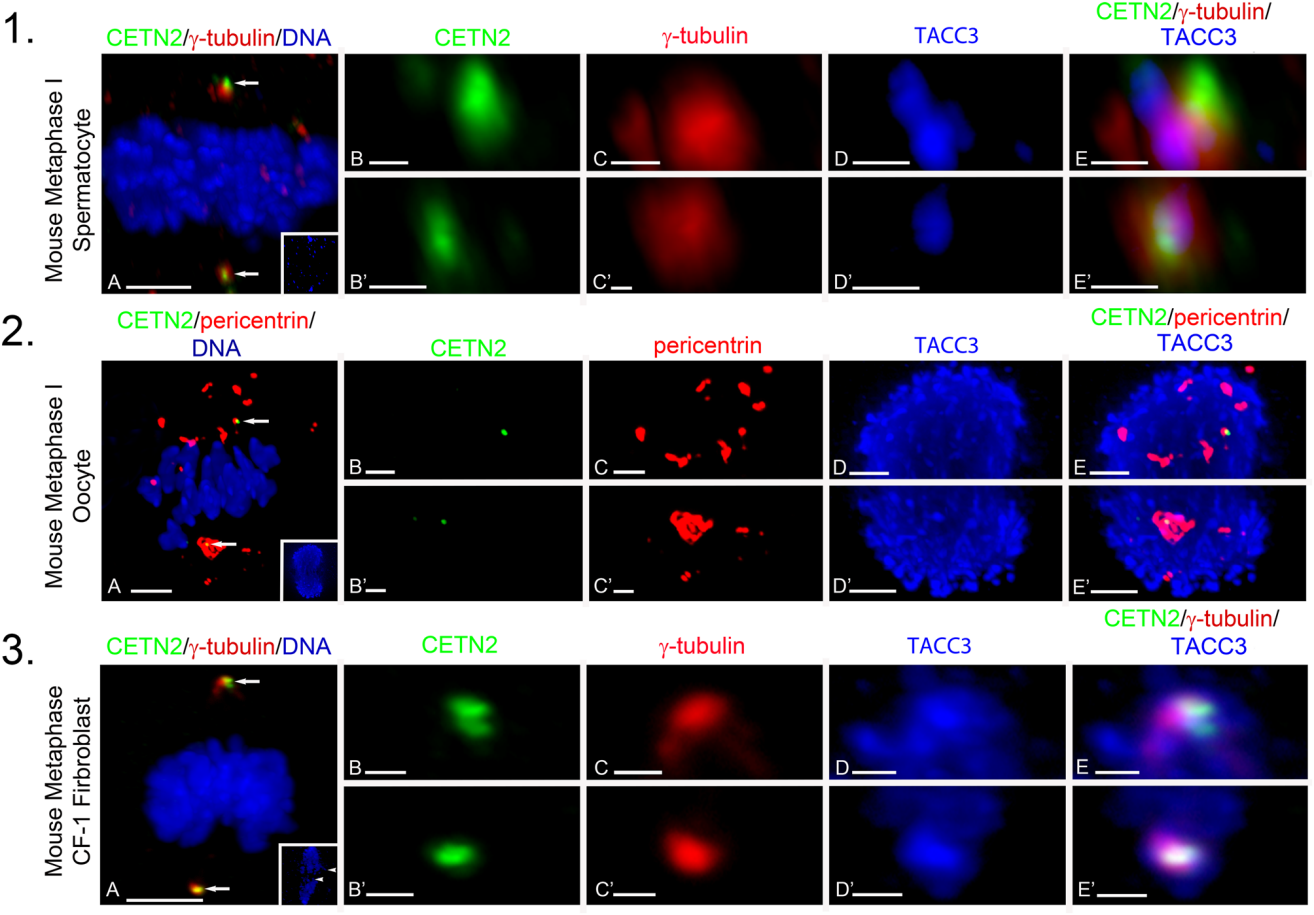
TACC3 is restricted to spindle poles in male meiosis spermatocytes as opposed to the spindle LISD in female oocytes or spindle pole/kinetochore microtubules in fibroblast cells. *Panel 1:* A metaphase-I mouse GFP CETN2-expressing spermatocyte (**A**: green, arrows) immunolabeled with γ-tubulin (A: red) and DNA (A: blue). Inset: TACC3 spindle pole immunolabeling (blue). (**B**–**D**) (upper pole) and (**B’**–**D’**) (lower pole) details; GFP CETN2 centrioles (**B**, **B’**: green), γ-tubulin (**C**, **C’**: red) and TACC3 (**D**, **D’**: blue). (**E**, **E’**): overlays. *Panel 2*: Metaphase-I mouse oocyte expressing GFP CETN2 (**A**: green, arrows) at the spindle poles immunolabeled with pericentrin (**A**: red) and DNA (**A**: blue). Inset: spindle LISD TACC3 (blue). (**B**–**D**) (upper pole) and (**B’**–**D’**) (lower pole) details; GFP CETN2 foci (**B**: green), spindle pole pericentrin MTOC (**C**: red), and the spindle LISD TACC3 (**D**: blue). (**E** and **E’**) Overlays. *Panel 3*: a mouse GFP CETN2-expressing CF-1 fibroblast cell (**A**: green, arrows) immunolabeled with γ-tubulin (**A**: red) and DNA (**A**: blue). Inset: TACC3 immunolabels the spindle poles and kinetochore microtubules (blue, arrowheads). (**B**–**D**) (upper pole) and (**B’**–**D’**) (lower pole) details; GFP CETN2 centrioles (**B**: green), γ-tubulin (**C**: red) and TACC3 (**D**: blue). (**E**, **E’**) Overlays. Panels 1A, 2A, and 3A scale bars = 5 µm; Panels 1, 2, 3 (**B**–**E**) and (**B’**–**E’**) scale bars: 1 µm.

The distinct TACC3 staining patterns observed between male and female meiotic and mitotic fibroblasts spindles is shown in Fig. 2. In meiotic spermatocytes, TACC3 often interfaces between the spindle pole γ-tubulin and the lateral surface of a centriole doublet but does not bind to intrapolar or kinetochore spindle microtubules nor the plus ends of microtubules near kinetochores (Fig. 2, panel 1). In meiotic oocytes, TACC3 is concentrated in the LISD extending beyond the spindle lattice and spindle microtubules, but without specific binding to spindle pole MTOC’s or maternal GFP CETN2-expressing foci (Fig. 2, panel 2). KTMTs strongly labeled with TACC3 in female meiotic spindles, but not microtubule plus-ends near kinetochores. In mitotic fibroblasts, TACC3 is bound to the spindle pole centrosomes and intrapolar as well as KTMT in the spindle proper, but typical not microtubule plus-ends near kinetochores.

**Figure 3:**
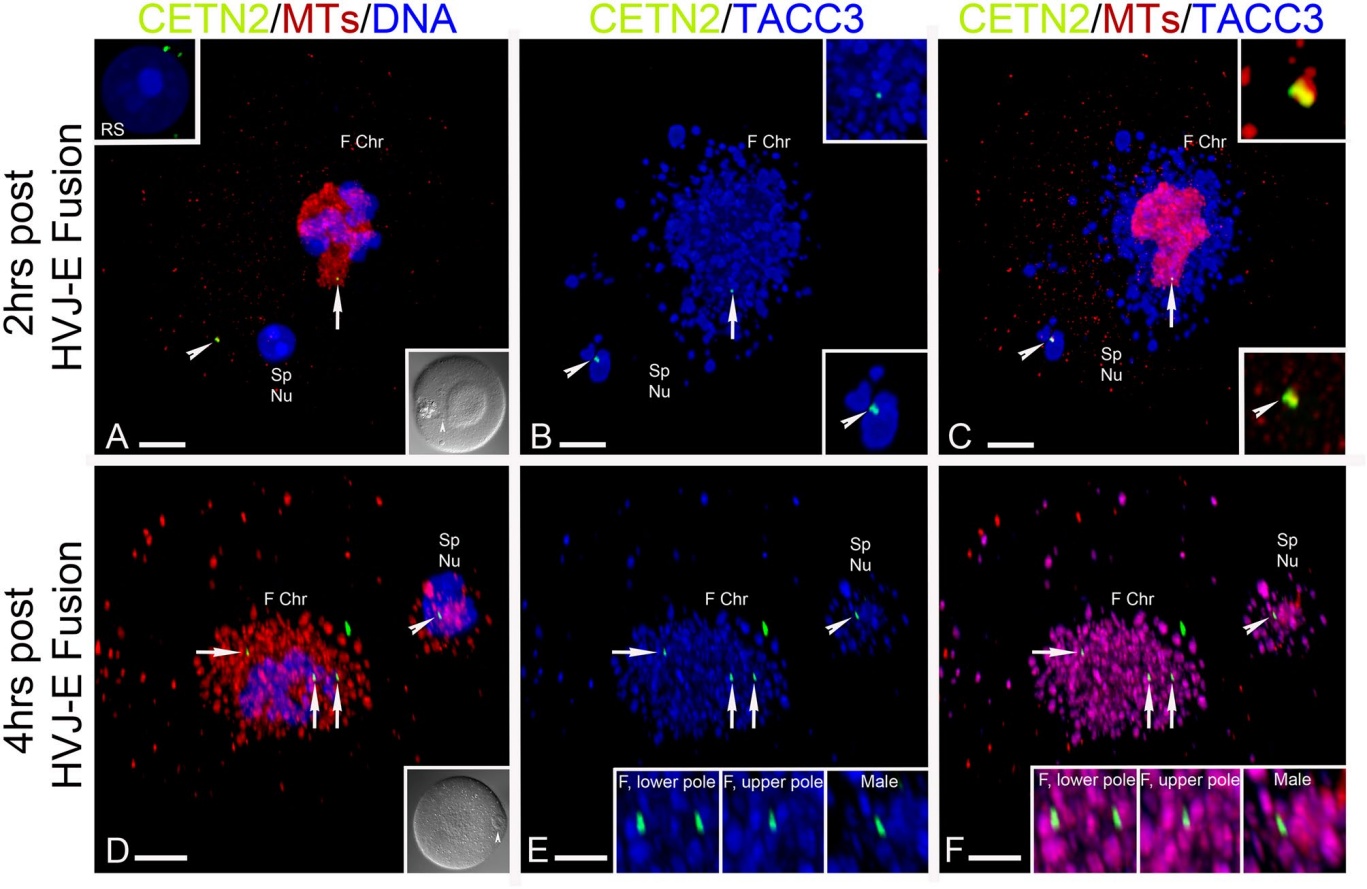
HVJ-E Sendai extract fusion of mouse round spermatids into GFP CETN2-expressing mouse oocytes demonstrates paternal TACC3 LISD organization at the incorporated paternal centrioles but sparse paternal microtubule assembly*.* (**A**–**C**) Prometaphase-I oocyte (**A**): DNA, F Chr; blue) 2-h post Sendai-extract fusion with a round spermatid (**A**: DNA, blue). TACC3 LISD condensates (**B**, **C**: blue) congress around and bind the assembling female meiotic spindle microtubules (**A**, **C**: red) harboring a single spindle pole GFP CETN2 expressing foci (**A**–**C**: green, arrow). The incorporated GFP CETN2-expressing male spermatid centrioles (**A**–**C**; green, arrowheads) also have TACCs LISD condensates engulfing them (**B**, **C**: blue, arrowheads) but without microtubule assembly (**A**, **C**: red). (**A**) Upper inset: a pre-fusion round spermatid (blue, DNA) expressing GFP CETN2 (green) but no TACC3 (blue). (**A**) lower inset: DIC image confirming spermatid:oocyte fusion (arrowhead). (**B**, **C**) upper insets; details, female TACC3 LISD condensates (**B**) and microtubule organization (**C**) at the GFP CETN2-expressing foci (**B**, **C**: green, arrow). (**B**, **C**) Lower insets: details, paternal TACC3 LISD condensates (**B**, blue, arrowhead) with no microtubules (**C**, red, arrowhead) at the incorporated paternal centrioles (**B**, **C**: green). (**D**–**F**) GFP CETN2-expressing spermatocyte (**D**: lower inset, arrowhead; DIC): GFP CETN2-expressing mouse GV oocyte dyad 4-h post-fusion. The female bivalents condense (**A**–**C**: F Chr; blue) as spindle microtubules assemble (**D**, **F**: red) with a single GFP CETN2-expressing maternal centriole foci (**D**–**F**: green, arrows) at one pole. Expansive TACC3 LISD condensates assembles around the developing spindle (**E**, **F**: blue). The incorporated spermatid expresses GFP CETN2 centrioles (**D**–**F**: green, arrowheads) near the male decondensing sperm chromatin (**D**: Sp Nu, blue) but shows sparse microtubule assembly (**D**, **F**: red). Extensive TACC3 LISD condensates (**E**, **F**: blue) surround and bind the poorly organized spindle microtubules at the paternal DNA (**E** and **F** insets: details, TACC3 (blue), microtubules (**F**: red), and GFP CETN2-expressing foci (**E**, **F**: green) at female (**F**) and male spindle poles. All images: GFP CETN2-expressing oocytes:spermatid fusions immunolabeled for microtubules (red; **A**, **C**, **D**, **F**), TACC3 (blue: **B**, **C**, **E**, **F**) and DNA (**A**, **D**: blue). F Chr, female chromosomes; Sp Nu, sperm nucleus; F, female. Scale bars = 10 µm.

The divergent patterns of TACC3 and cKAP5/chTOG detection observed in meiotic or mitotic spindles suggest that these proteins may have distinctly different roles in supporting spindle organization, stabilization, and functions. To explore this further, spermatocytes were fused into a mouse GV-stage meiotic oocyte using HVJ- E Sendai extract fusion protein to investigate how TACC3 protein would organize at the paternal centrosome within the maternal cytoplasmic environment. After confirming fusion by differential interference microscopy (DIC; Fig. 3A,D), oocytes resumed meiosis by washout of dbcAMP. Round spermatids (RS) mostly silence GFP CETN2 expression and never immunolabel with TACC3 antibody (Fig. 3A, upper inset; see Suppl Fig. S2, panel 3). By 2 h post fusion, the incorporated RS showed GFP CETN2-expressing centrioles in the oocyte’s cytoplasm surrounded by large maternal TACC3 condensates, but no microtubule organization as observed around the condensing female chromosomes and assembling TACC3 LISD (Fig. 3A–C). By 4-h post- oocyte fusion, weak disorganized microtubules surrounded the paternal GFP CETN2-expressing centrioles and slightly decondensed sperm nucleus as extensive maternal TACC3 condensates surrounded the paternal DNA and co-immunostained the sparse microtubules, similar in appearance to TACC3 staining of the female spindle microtubules and LISD (Fig. 3D–F). Thus, spermatocytes that typically restrict TACC3 binding to their centrosomes assemble TACC3 LISD condensates around the paternal DNA when introduced into the maternal cytoplasmic environment, despite poorly organized microtubules and incomplete DNA decondensation.

**Figure 4:**
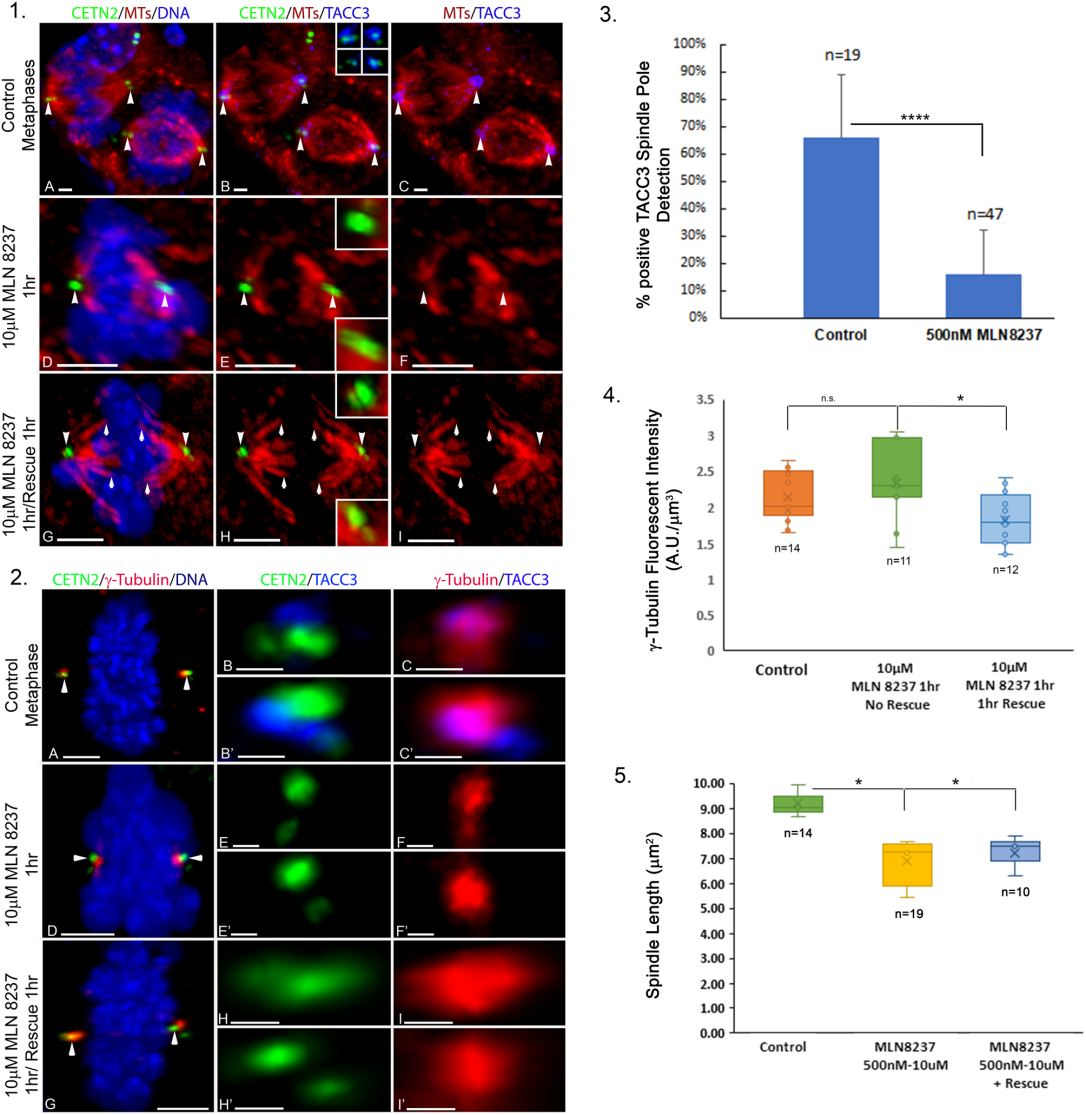
GFP CETN2-expressing spermatocytes exposed to MLN 8237 Aurora A kinase inhibitor significantly depleted spindle pole TACC3, inducing spindle microtubule abnormalities, reduced spindle lengths, and misaligned chromosomes but not γ-tubulin spindle pole intensity. *Panel 1*: GFP CETN2-expressing spermatocytes (green, arrowheads: **A**, **B**, **D**, **E**, **G**, **H**) in untreated controls (**A**–**C**), 10 µM MLN 8237 anti-Aurora A kinase inhibitor (**D**–**F**) or after rescue from 10 µM MLN 8237 exposure (1 h; **G**–**I**). MLN 8237 1 h exposure removed spindle pole TACC3 (blue; **E**, **F**), disrupted bipolar spindle organization (red; **E**, **F**), and chromosome alignment (**D**: blue) compared to untreated spermatocytes (**A**–**C**), but recovery experiments did not restore spindle pole TACC3 (**H**, **I**; blue) or chromosome alignment (**G**: blue) despite partial spindle kinetochore microtubule recovery (**H**, **I**: red, arrows). Insets: (**B**, **E**, **H**) GFP CETN2-expressing centrioles (green), microtubules (red) and spindle pole TACC3 (blue). *Panel 2*: MLN 8237 impact on spindle pole γ-tubulin. GFP CETN2-expressing spermatocytes (**A**, **D**, **G**: green, arrowheads) in untreated controls (**A**–**C’**), 10 µM MLN 8237 anti-Aurora A kinase inhibitor (1 h; **D**–**F’**) and after rescue from 10 µM MLN 8237 exposure (1 h; **G**–**I’**). MLN 8237 depleted spindle pole TACC3 (**E**, **E’**; blue) induced chromosome misalignment (**D**: DNA, blue), but not γ-tubulin spindle pole detection (**F**, **F’**: red) compared to control spermatocytes (**A**–**C’**). MLN 8237 recovery experiments did not restore spindle pole TACC3 (**H**, **H’**, **I**, **I’**: blue), DNA realignment (**G**: DNA, blue) or significantly impact γ-tubulin detection (**G**, **I**, **I’**: red). *Panel 3*: Analysis of spermatocyte MLN 8237 exposure showed the lowest concentrated tested (500 nM) significantly reduced spindle pole TACC3 compared to controls (****; *p* < 0.00009). *Panel 4*: γ-tubulin spindle pole intensity measured at the highest MLN 8237 concentration tested (10 μM; 1 h) did not impact γ-tubulin compared to controls (ns; *p* < 0.1485) but slightly reduced γ-tubulin intensity compared to controls after 1 h rescue (*; *p* < 0.0113), suggesting poor recovery from MLN 8237pleiotropic effects during meiosis. *Panel 5:* Spermatocyte spindle pole-to-pole lengths were significant from controls after 500 nM MLN 8237 exposure (*; *p* < 0.0152) or 1 h rescue experiments (*; *p* < 0.0158). All scale bars = 5 µm.

To better understand the role of TACC3 in spermatocyte meiotic spindle assembly and maintenance, the small molecular AURKA inhibitor MLN 8237 was used to disrupt spindle pole TACC3 in spermatocytes (Fig. 4). Control spermatocytes had spindle pole TACC3 only (Fig. 4, panel 1 A–C). At 10 µM for 1 h, MLN 8237 abolished spindle pole TACC3, disrupted normal bipolar spindle microtubule organization, and increased chromosome misalignment (Fig. 4, panel 1 D–F). Washout experiments from MLN 8237 exposure did not show spindle pole TACC3 recovery, although sparse spindle and kinetochore microtubules unfocused at the poles was apparent (Fig. 4, panel 1 G–I). No chromosome realignment was observed. 500 nM MLN 8237 showed significant spindle pole TACC3 loss compared to controls (Fig. 4, panel 3; ****; *p* = 0.00009) without spindle pole TACC3 recovery after washout experiments (Fig. 4, panel 1 H, I; 0/15; n = 3 trials).

We also investigated impacts on spindle pole γ-tubulin after MLN 8237 exposure to disrupt TACC3 in spermatocytes (Fig. 4, panel 2 A–C). 10 µM MLN 8237 treatment for 1 h showed loss of spindle pole TACC3 with poorly aligned meiotic chromosomes (Fig. 4, panel 2 D–F) but a measurable increase in spindle pole γ-tubulin detection based on fluorescent intensity measurements, though not statistically significant compared to controls (Fig. 4, panel 4; ns; *p* < 0.1485). After MLN 8237 washout for 1 h, spindle pole γ-tubulin fluorescent intensity was significantly reduced below control and MLN 8237 treated levels (Fig. 4, panel 4; *; *p* < 0.0113) despite no spindle pole TACC3 recovery (Fig. 4, panel 2 G–I). Spindle pole-to-pole length was also marginally reduced after MLN 8237 exposure compared to controls (~ 16%; Fig. 4, panel 5; *; *p* < 0.01530) and did not fully recover following drug washout for 1 h (Fig. 4, panel 5: *; *p* < 0.0158).

**Figure 5:**
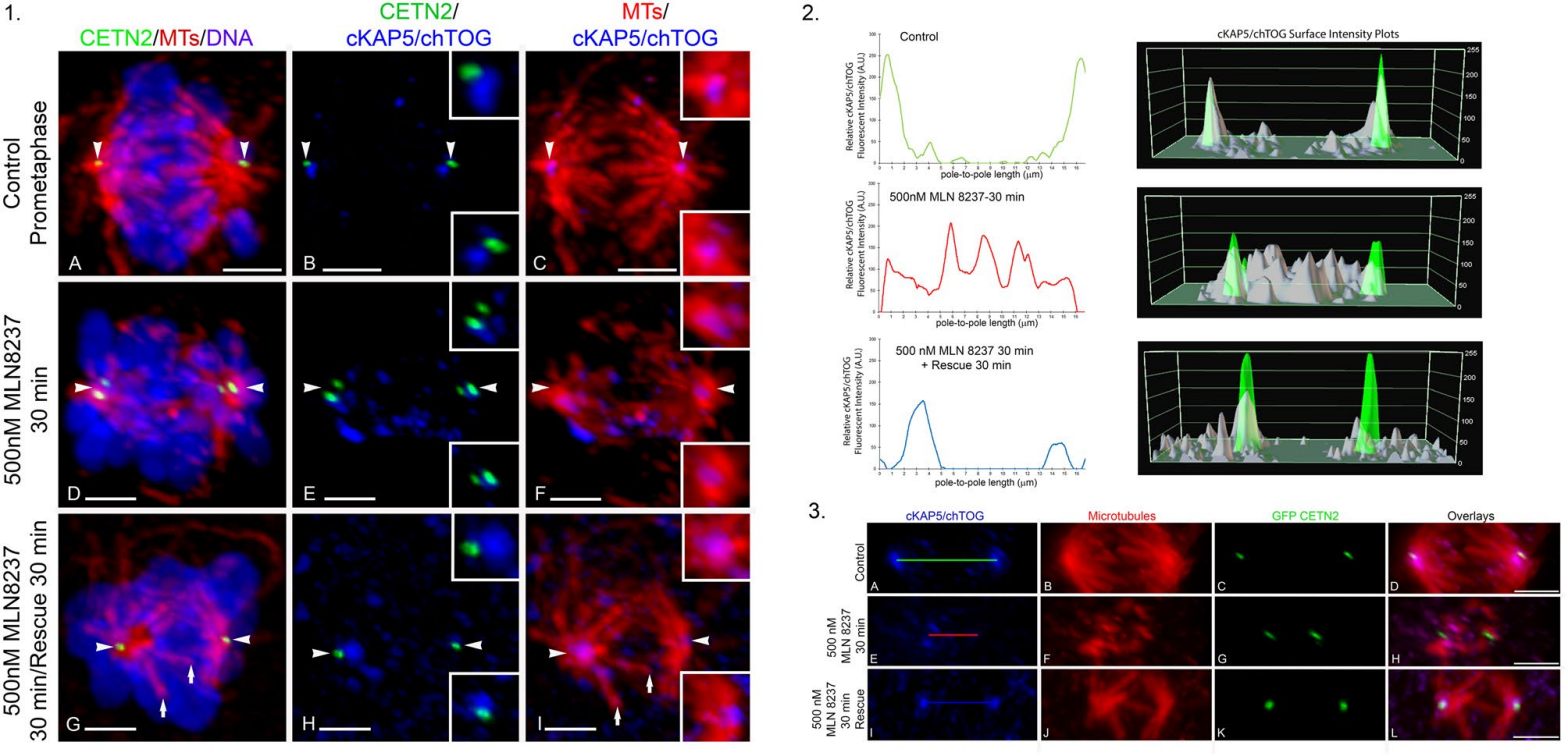
Unlike TACC3, GFP CETN2-expressing spermatocyte spindle pole cKAP5/chTOG is not entirely removed after exposure to MLN 8237 Aurora A kinase inhibitor despite spindle microtubule disorganization and chromosome misalignment. *Panel* 1: (**A**–**C**) Control GFP-CETN2-expressing spermatocytes (**A**, **B**: green) with spindle pole cKAP5/chTOG (**B**, **C**: blue) on a bipolar spindle (**A**, **C**: red, microtubules; **A**: blue, DNA). (**D**–**F**) 500 nM MLN 8237 (30-min) reduced visible spindle pole cKAP5/chTOG (**E**, **F**: blue; insets, details) with increased intra-spindle cKAP5/chTOG detection, though not on abnormal assembled spindle microtubules (**D**, **F**: red, microtubules; **D**: blue, DNA). (**G**–**I**) 500 nM MLN 8237 rescue experiments (30-min) showed spindle pole cKAP5/chTOG recovery (**H**, **I**: blue; insets, details) at the GFP CETN2-expressing centrioles (**G**, **H**: green) as spindle kinetochore microtubules reassembled (**G**, **I**: red, arrows) but chromosome remained unaligned (**G**: DNA, blue). *Panel 2*: cKAP5/chTOG relative fluorescent intensity (left) and surface intensity plots (right) for control (upper panels), 500 nM MLN 8237 exposed (middle panels) and 500 nM MLN 8237 with a 30 min rescue (lower panels). Control spermatocytes show mostly spindle pole cKAP5/chTOG intensity. MLN 8237 exposure reduces spindle pole cKAP5/chTOG and increased intra-spindle cKAP5/chTOG intensities. Rescue experiments partially re-establishes spindle pole cKAP5/chTOG while reducing intra-spindle intensities. Surface intensity plots: GFP CETN2 centriole plot (green) overlaid on cKAP5/chTOG plot (grey). *Panel 3*: images for intensity measurements shown in panel 2. Control (**A**–**D**), 500 nM MLN 8237 exposed (**E**–**H**) and rescue experiments from 500 nM MLN 8237 treatment (**I**–**L**). (**A**, **E**, **I**) bars, pole-to-pole measurements for intensity plotting. (**D**, **H**, **L**) overlays of cKAP5/chTOG (blue), microtubules (red) and GFP-CETN2 centrioles (green). MLN 8237 exposure disrupts normal bipolar spindle configuration (**F**; red, microtubules), reduces spindle length (**G**: green, GFP CETN2 centrioles), and increases cKAP5/chTOG (**E**: blue) within the spindle but not binding on remaining microtubules (**H**: overlay). Rescue experiments partially reverses spindle pole cKAP5/chTOG intensity while reducing intra-spindle protein intensity. All images: GFP CETN2-expressing centrioles (green) immunolabeled for YL1/2 microtubules (red), cKAP5/chTOG (blue), except panel 1 (**A**, **D**, **G**) (blue, DNA). Scale bars = 5 μm.

cKAP5/chTOG detection was investigated after MLN 8237 exposure to disrupt spindle pole TACC3 (Fig. 5). Control spermatocytes showed bipolar spindles with mostly aligned chromosomes and cKAP5/chTOG tightly adjacent to the GFP CETN2-expressing centrioles (Fig. 5A–C). Exposure to 500 nM MLN 8237 for 30 min significantly reduced spindle microtubule density and organization, elicited widely scattered meiotic chromosomes and reduced, but did not eliminate, spindle pole cKAP5/chTOG detection. cKAP5/chTOG detection increased within the spindle lattice after MLN 8237 exposure but did not directly bind on the remaining spindle microtubules (Fig. 5D–F). Fluorescent and surface plot intensity measurements on MLN 8237 exposed spermatocytes dramatically show spindle pole cKAP5/chTOG reduction along with the rise within the intra-spindle lattice without significant cKAP5/chTOG spindle microtubule binding (Fig. 5, panel 2 and 3). Rescue from MLN 8237 exposure for 30 min increased spindle pole cKAP5/chTOG detection with improved spindle microtubule organization, including KTMT assembly, but not chromosome alignment (Fig. 5, panel 1 G–I; short arrows, KTMTs). Interestingly, fluorescent and surface plot intensity measurements showed significant decreased of intra-spindle cKAP5/chTOG protein concomitant with an increase of cKAP5/chTOG at the spindle poles (Fig. 5, panels 2 and 3).

**Figure 6:**
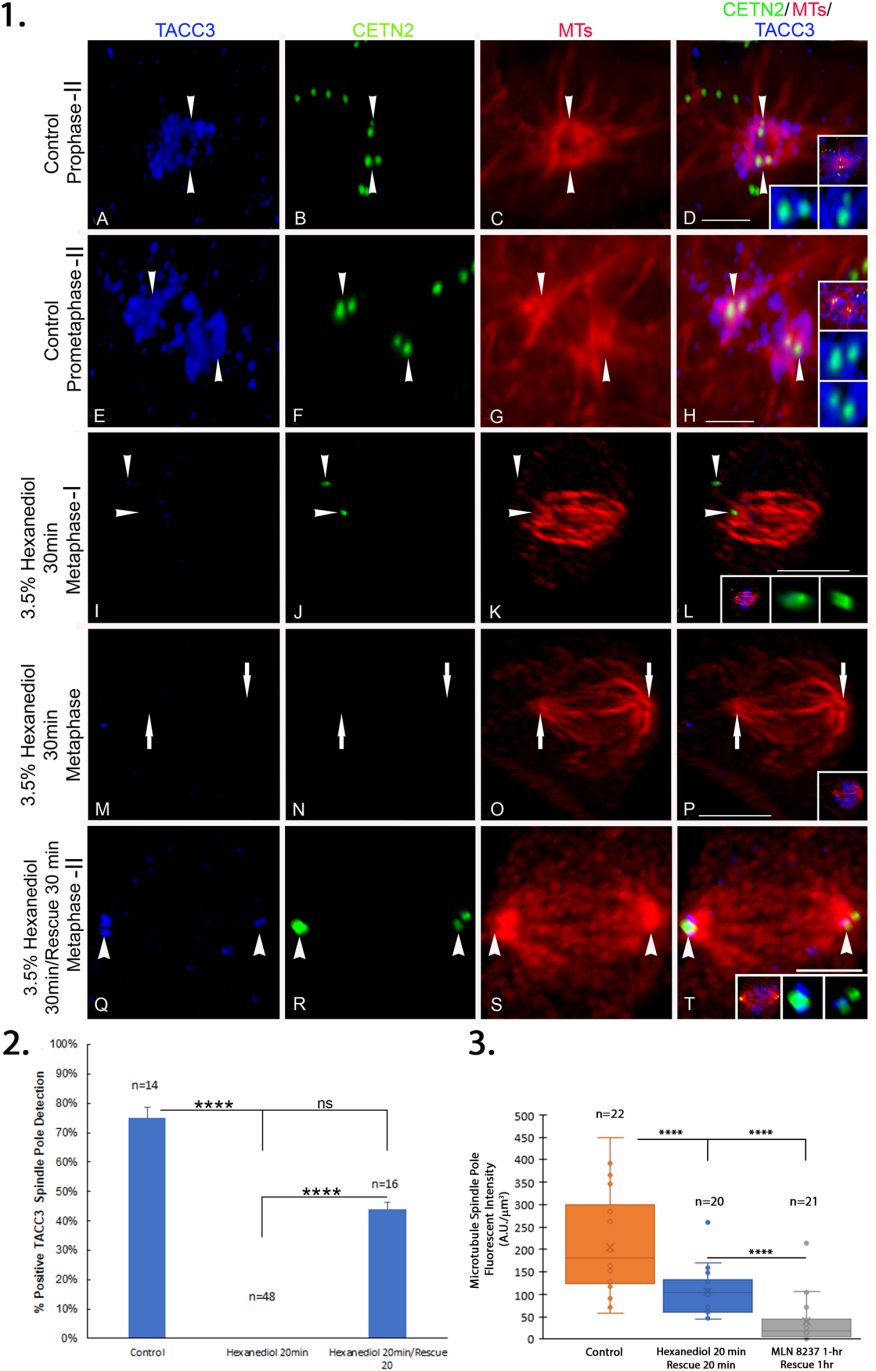
3.5% 1,6 hexanediol exposure depletes spindle pole TACC3 in GFP CETN2-expressing spermatocytes resulting in meiotic spindle microtubule malformation, centriole abnormalities, and chromosome misalignment. *Panel 1*: TACC3 detection (**A**, **E**, **I**, **M**, **Q**) in GFP CETN2-expressing (**B**, **F**, **J**, **N**, **R**) meiotic spermatocytes (**C**, **G**, **K**, **O**, **S**; microtubules, red). Control untreated spermatocytes (**A**–**C** and **E**–**G**) show strong TACC3 spindle pole assembly (**A**, **E**: blue) marked by GFP-CETN2 centrioles (**B**, **F**: green, arrowheads) at assembling spindle microtubules (**C**, **G**: red, arrowheads). (**D**, **H**) Overlays of GFP CETN2 (green), MTs (red) and TACC3 (blue). Top insets: GFP CETN2 centrioles (green), microtubules (red), and DNA (blue). Bottom insets: details, GFP CETN2 centriole (green) and TACC3 (blue). (**I**–**K**) 3.5% 1,6-hexanediol spermatocyte exposure for 30 min removes TACC3 detection at spindle poles (**I**, **M**, **Q**), may reduce spindle microtubule density and organization (**K**, **O**, **S**: microtubules, red), disrupts GFP CETN2-expressing centrioles spindle pole localization (**N**: green) or detection (**R**: green), and impacts normal chromosome spindle alignment (left insets: **L**, **P**; lower inset, **T**: DNA, blue) (**L**, **P**, **T**): overlays of GFP CETN2 (green), microtubules (red) and TACC3 (blue). (**L** and **P**), middle and right insets: GFP CETN2 (green) and TACC3 (blue). Scale bars = 5 µm. *Panel 2*: TACC3 detection at the spindle poles of GFP CETN2-expressing spermatocytes exposed to 3.5% 1,6-hexanediol exposure for 30 min shows complete spindle pole removal of TACC3 (****; *p* < 0.0). Rescue from 1,6- hexanediol for 20 min before fixation showed a partial recovery not statistically significant from control cells (ns.; *p* < 0.235148) but significant from hexanediol exposure (****; *p*, 0.000004). *Panel 3*: measured fluorescent intensity of spindle pole microtubule reassembly is significantly greater in spermatocytes recovered from 3.5% 1,6-hexanediol exposure for 20 min that can restore TACC3 to the centrosomes compared to 10 µM MLN 8237 exposed spermatocytes recovered for 1-h that do not restore centrosomal TACC3 (****; *p* < 0.000034), although neither treatment approaches full spindle microtubule intensity of control spermatocytes (control vs hexanediol rescue: ****; *p* < 0.0005; control vs MLN 8237: ****; *p* < 0.000001).

The liquid-phase condensate disruptor 1,6-hexanediol^51,52^ disrupts TACC3 in the LISD of female meiotic oocytes but did not impact meiotic spindle pole microtubule TACC3 detection^47^. Here, 3.5% 1,6-hexanediol exposure on spindle pole TACC3 localization in male spermatogenic cells was explored (Fig. 6). Control prophase-I meiotic spermatocytes showed observed spindle pole and centriole TACC3 detection (Fig. 6, panels 1 A–H). 3.5% 1,6-hexanediol for 30 min abolished spindle pole TACC3 inducing abnormal male meiotic metaphase spindle phenotypes having misplaced or no GFP CETN2-expressing centrioles, poor bipolar spindle organization with reduced spindle microtubule density and misaligned chromosome (Fig. 6E–P). In 30 min washout recovery experiments, significant spindle pole TACC3 recovery occurred after hexanediol removal compared to hexanediol exposure (Fig. 6, panel 2; ****; *p* < 0.000004), with improved bipolar spindle pole microtubule organization but not chromosome alignment (Fig 6Q–T). Analysis of microtubule fluorescent intensity at the spindle poles following recovery from 1,6-hexanediol was significantly different from measured microtubule fluorescent intensity following a 1-h recovery from MLN 8237 AURKA inhibitor, but not fully when compared against control spermatocytes (Fig. 6, panel 3). This greater density of spindle pole microtubules observed after washout of 1,6-hexanediol may reflect the ability to restore spindle pole TACC3 compared to MLN 8237 recovered spermatocytes that do not reassemble spindle pole TACC3.

**Figure 7:**
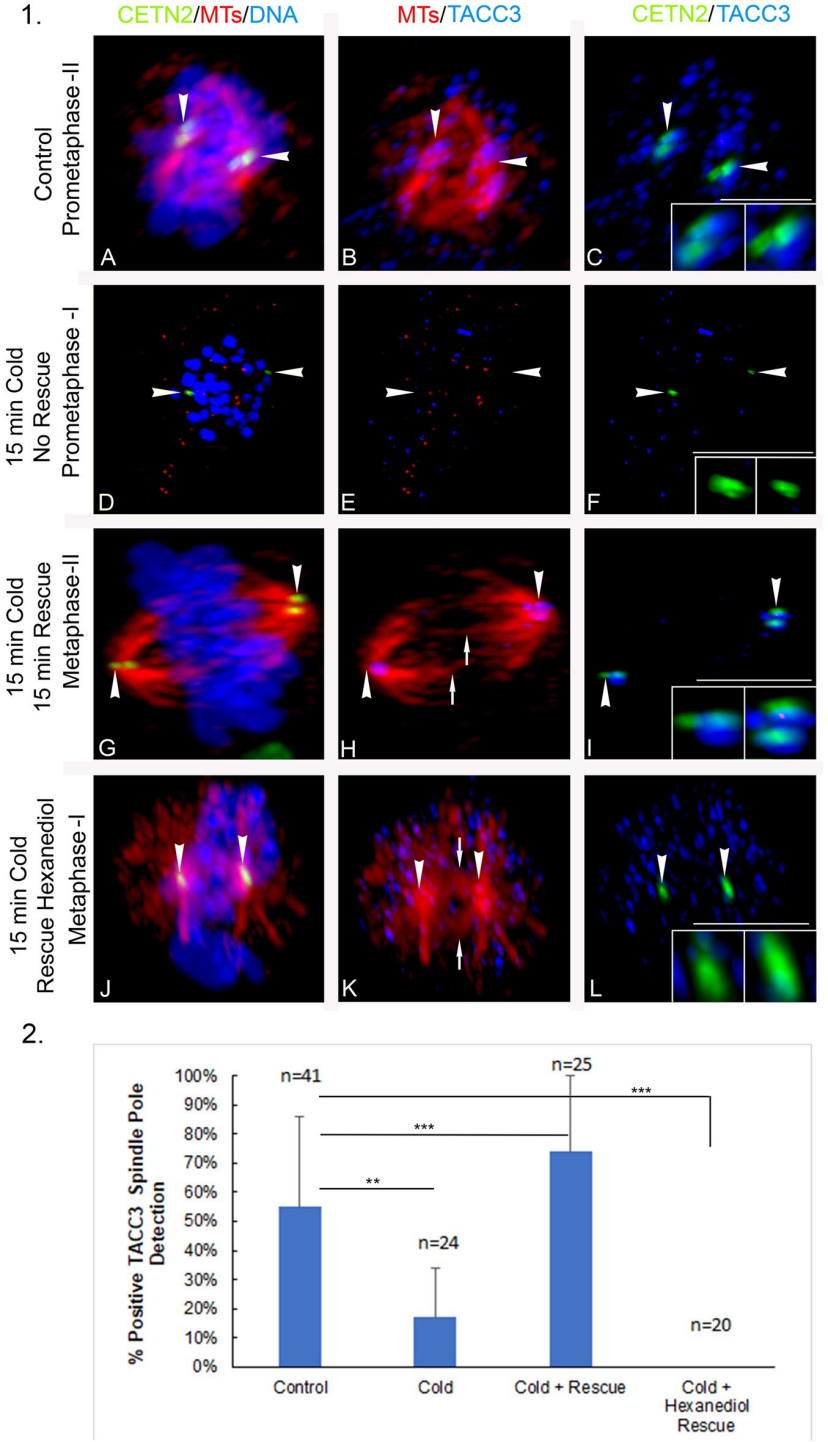
Cold depolymerization of spermatocyte meiotic spindle microtubules removes spindle pole TACC3 with impacts on chromosome alignment, but recovery experiments show rescue of spindle pole TACC3, microtubule reassembly and chromosome realignment unless in the presence of 3.5% 1,6-hexanediol that blocks spindle pole TACC3 but not initial spindle pole microtubule polymerization. *Panel 1*: (**A**–**C**) control prometaphase-II spermatocyte showing bipolar spindle assembly (**A**, **B**: microtubules, red), aligning chromosomes (**A**: DNA, blue), and GFP CETN2 centrioles (**A**, **C**: green, arrowheads) with spindle pole TACC3 (**B**, **C**: blue, arrowheads). (**C**) insets: details, GFP CETN2 centrioles (green) and TACC3 (blue). (**D**–**F**) Prometaphase-I spermatocyte exposure to cold conditions for 15 min initiates spindle microtubule disassembly (**D**, **E**: red), misaligned chromosomes (**D**: DNA, blue), and loss of spindle pole TACC3 (**E**, **F**: blue, arrowheads) marked by GFP CETN2 centrioles (**D**, **F**: green; arrowheads). (**F**) Insets: details, GFP CETN2 centrioles (green) and TACC3 (blue). (**G**–**I**) A metaphase-II spermatocyte exposed for 15 min to cold conditions plus 15 min rescue in warm conditions shows bipolar spindle reassembly (**G**, **H**: microtubules, red) with kinetochore microtubules (**H**: red, arrows), realigning chromosomes (**G**: DNA, blue) and spindle pole TACC3 (**H**, **I**: blue, arrowheads) at the GFP CETN2 centrioles (**G**, **I**: green, arrowheads). Assembling spindle kinetochore microtubules do not label with TACC3 (**H**: blue, arrows). (**I**) insets: details, GFP CETN2 centrioles (green) and TACC3 (blue). (**J**–**L**) A metaphase-I spermatocyte exposed for 15 min to cold conditions plus 15 min rescue in warm conditions in the presence of 3.5% 1,6-hexanediol shows extensive cytoplasmic TACC3 without binding at the GFP CETN2 centrioles (**J**, **L**: green, arrowheads), significant microtubule assembly from both spindle poles (**J**, **K**: red) with kinetochore microtubules (**K**: red, arrows) and still misaligned chromosomes (**J**: DNA, blue). The reassembling kinetochore microtubules do not label with TACC3 (**K**: blue, arrows). (**L**) Insets: details, GFP CETN2 centrioles (green) and TACC3 (blue). Scale bars = 5 µm. *Panel 2*: graphic analysis of TACC3 spindle pole detection shows significant deviation from non-treated controls following cold exposure (**; *p* < 0.01147), cold with 15 min rescue (***; *p* < 0.006156), and cold with 15 min rescue in presence of 3.5% 1,6-hexanediol (***; *p* < 0.01419).

To further explore spindle pole TACC3 role in spindle microtubule assembly in spermatocytes, microtubule depolymerization and recovery experiments in the presence of 1,6-hexanediol were performed. Spermatocytes were placed in ice-cold media for 15 min to depolymerize spindle microtubules, then recovered in warm culture media (34̊C) with or without 3.5% 1,6-hexanediol before examining spindle pole TACC3 and spindle microtubules in fixed samples (Fig. 7). Compared to controls, 15 min in ice-cold media completely depolymerized spindle microtubules, significantly reduced spindle pole TACC3 detection, and induced chromosome misalignment (Fig. 7, panel 1 D–F; panel 2; second bar; **; *p* < 0.011470). Rescue experiments in warm culture media for 15 min restored spindle pole TACC3 even greater than in control spermatocytes (Fig. 7, panel 1 G–I; panel 2, third bar; ***; *p* < 0.006156) and assembled robust intrapolar and kinetochore microtubules with aligned meiotic chromosomes, although the spindle microtubules did not label with TACC3 (Fig. 7, panel 1H). When recovery experiments were performed for 15 min in the presence of 3.5% 1,6-hexanediol, strong centrosomal astral microtubule assembled from both poles, despite no spindle pole TACC3 detection or chromosome realignment (Fig. 7, panel 1 J–L). 3-dimensionnal image rotations of rescued spermatocytes in the presence of 1,6-hexanediol showed abundant cortical and cytoplasm TACC3 foci after 15 min in warm culture conditions without specific binding to spindle pole centrioles or reformed spindle microtubules (Fig. 7, panel 1 K–L; panel 2, 4th bar; ***; *p* < 0.001419). Thus, spindle pole TACC3 may not be required for initial microtubule polymerization from spindle pole centrosomes, but bipolar organization with proper chromosome alignment may require TACC3.

Further experiments explored the role of microtubule dynamics on spindle pole TACC3 and cKAP5/chTOG in male meiotic spindles (Suppl Fig. S5). Meiotic spermatocytes exposed to 10 µM nocodazole microtubule depolymerization agent for 30 min showed cells without assembled microtubules but retained spindle pole TACC3 and cKAP5/chTOG detection in close association with GFP CETN2-exrpessing centrioles (Suppl Fig. S5; panel 1 D–F and panel 2 D–F). Likewise, 30 min in 10 µM paclitaxel, a microtubule stabilizing agent that significantly increases spindle microtubules, did not remove spindle pole TACC3 or cKAP5/chTOG detection, but appeared to reduce tight centriole association (Suppl Fig. S5, panel 1 G–I and panel 2 G–I). Analysis of spindle pole TACC3 and cKAP5/chTOG after nocodazole or paclitaxel application did not show significant reduction in these spindle pole proteins compared to untreated control spermatocytes (Suppl Fig. S5, panel 3). These results imply that microtubule dynamics may not play a significant role in spindle pole TACC3 or cKAP5/chTOG retention in male meiotic spermatocytes.

**Figure 8:**
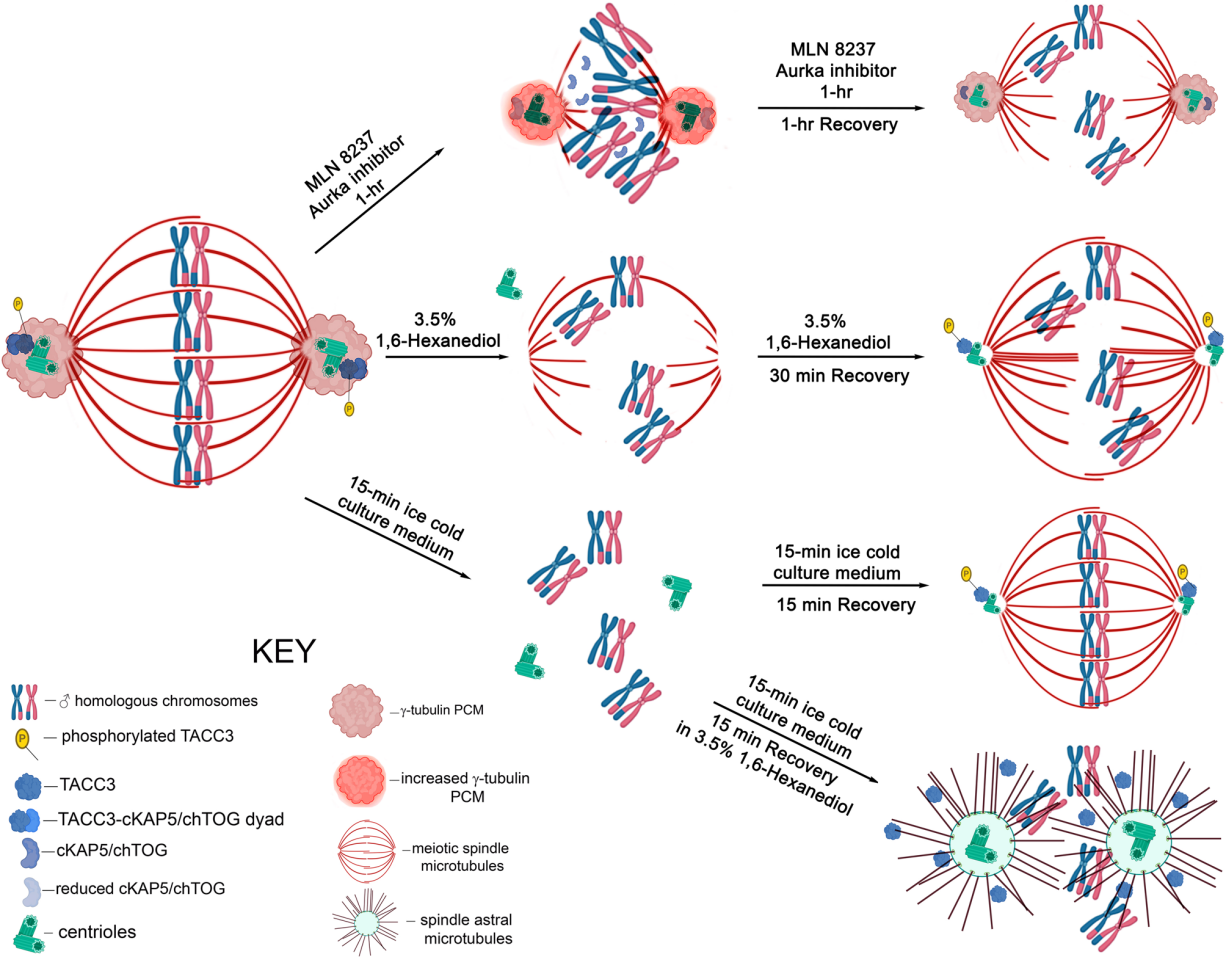
Male meiotic spindle organization is lost after MLN 8237 Aurora A kinase inhibitor and 1,6-hexanediol disruption of spindle pole TACC3. Meiotic spermatocytes have anastral bipolar metaphase spindles with aligned chromosomes and canonical centrosomes at both spindle poles. TACC3 and cKAP5/chTOG function as a dyad at the poles, strongly interfacing between the γ-tubulin pericentriolar material and centriole doublets. TACC3 phosphorylation is almost certainly necessary to bind the dyad to spindle poles^34^. Spindle pole TACC3 appears required for male meiotic spindle bipolarity, as exposure to MLN 8237 Aurora A kinase inhibitor (top panel) or hexanediol (middle panels) shows reduce, shorter, and highly disarrayed spindle microtubules with misaligned chromosomes. Spindle pole organization is also impacted by TACC3 disruption, with MLN 8237 exposure slightly enhancing spindle pole γ-tubulin intensity but reducing spindle pole cKAP5/chTOG and shifting the protein to the spindle lattice, although without significant binding on the remaining intact microtubules. 1,6-hexanediol exposure either displaces centrioles from the spindle pole or removes centriole GFP CETN2-expression at the disorganized bipolar spindles. Interestingly, TACC3 does not appear required for spindle pole microtubule assembly, as recovery from MLN 8237 AURKA inhibitor did not block partial microtubule recovery, including assembly of kinetochore microtubules, despite no detection of spindle pole TACC3 after washout. Conversely, rescue from 1,6-hexanediol did restore centriole-associated spindle pole TACC3 with better spindle pole microtubule organization. However, neither recovery from MLN 8237 inhibitor nor 1,6-hexanediol instituted chromosome realignment to the spindle equator. Cold microtubule disassembly and regrowth experiments in the presence of 1,6-hexanediol (lower panels) reinforces that spindle pole TACC3 is not required for microtubule assembly as spindle pole astral microtubules reassemble without TACC3 detection at the centrioles despite abundance of TACC3 protein detection in the cytoplasm. Here, TACC3 does not bind to centrioles perhaps because it cannot be phosphorylated to target the centrosomes in the presence of 1,6-hexanediol. AURKA: Aurora A kinase.

Figure 8 summarizes our major findings on TACC3 and cKAP5/chTOG detection in spermatocytes and how disrupting or recovery of spindle pole TACC3 impacts spindle organization and integrity in male meiotic spindles.

## Discussion

In a recent important study of male meiosis, spermatocytes from AURKA conditional knockout mouse lacked centriole colocalized TACC3 despite maintaining normal cellular TACC3 protein levels, underscoring the importance of Aurora A kinase in TACC3 centrosome recruitment to male meiotic spindle poles. TACC3 disruption appeared to induce misoriented chromosomes in meiosis-I spermatocytes^18^. But no information has yet been reported on the role(s) of TACC3 and cKAP5/chTOG in spermatocyte meiotic spindle assembly and stabilization during critical stages of chromosomal divisions.

Here, spermatocytes from a specialized mouse mutant that directly expresses GFP centrin-2 in centrioles to mark the poles were immunostained with antibodies to detect microtubules, γ-tubulin PCM, TACC3 and cKAP5/chTOG^17^. We show TACC3 and cKAP5/chTOG proteins appear exclusively at the meiotic spindle poles in mouse spermatocytes, often bound between centriole doublets and the γ-tubulin PCM (Figs. 1, 2). Surprisingly, unlike observations in mitotic fibroblast or meiotic oocyte spindles, meiotic spermatocytes did not show TACC3 or cKAP5/chTOG detection on intrapolar or kinetochore spindle microtubules, nor did these proteins appear at the plus-ends of microtubules near kinetochores as reported in somatic and cancer cells (Fig. 1, panels 2 and 4 cells)^38–40,50^. No centrosomal TACC3 detection was observed in non-meiotic spermatocytes (Suppl Fig. S2). Thus, TACC3 and cKAP5/chTOG are uniquely spermatocyte meiotic spindle pole proteins in male mice, markedly distinct from the reported locality of these proteins in the LISD and spindle microtubules of female mammalian meiotic oocytes (Suppl Figs. S3, S4)^41–47^ or in multiple spindle sites in mouse GFP CETN2-expressing fibroblasts (Figs. 1, 2) ^rev,^^28^.

No meiotic male spindle LISD was observed in mouse spermatocytes as observed in female oocytes (Fig. 1)^47^. We were curious if a male spermatocyte centrosome introduced into the female oocyte cytoplasm could attract TACC3 LISD condensates to the paternal centrioles to assemble a bipolar spindle with aligned paternal chromosomes (Fig. 3). From fusions produced and culture in vitro, we first found extensive maternal TACC3 LISD aggregation surrounding the male centriole doublets, remarkably like TACC3 detected in male meiotic spindle poles (Fig. 1, panel 2). However, by 4-h post-fusion, the TACC3 LISD aggregates co-immunolabel with sparse, disorganized microtubules, not the unduplicated paternal centrioles, near the incomplete decondensed male chromosomes, a pattern like the LISD around female condensing chromosomes. The paternal centrosome is most likely inactive in spindle organization within the oocyte’s cytoplasm, consistent with observations showing that mouse oocytes repress centrioles and their activities during meiotic stages^14^. Thus, mouse spermatocytes introduced into maturing mouse oocytes assemble a TACC3 LISD condensate like female spindles, but the paternal LISD is insufficient to support a fully functioning paternal bipolar spindle, perhaps suggesting other significant spindle protein recruitment is required to complete proper spindle assembly.

In mitotic cells, siRNA TACC3 depletion studies severely impacted spindle pole astral microtubule assembly by interfering with γ-TuRC recruitment to the centrosomes. However, no major impact on spindle bipolarity was reported despite some reduced spindle microtubule density and misaligned chromosomes (Gergely et al.^29^; Singh et al.^25^; Rajeev et al.^26^). Meiotic spermatocytes exposed to the AURKA inhibitor MLN 8237 lost spindle pole TACC3 detection (Fig. 4 and 5; Wellard et al.^18^). Spermatocytes, which do not assemble spindle pole astral microtubules, showed significant reductions of meiotic spindle microtubule density, bipolar organization, pole-to-pole spindle length, and misaligned meiotic chromosomes after MLN 8237 depletion of TACC3. Aberrant spindle phenotypes observed in MLN 8237 exposed spermatocytes appeared more severe in comparison to human mitotic cells after TACC3 depletion^25,26,29^ but perhaps like oocyte meiotic spindles from AURKA or pericentrin knockout mice lacking the ability to assemble the spindle TACC3 LISD^45,46^.

Spermatocyte spindle pole TACC3 depletion by MLN 8237 did not fragment the PCM or produce supernumerary spindle poles, as reported in Aurora A RNAi depletion experiments in mitotic cells^53^. However, we observed a measurable increase in spindle pole γ-tubulin fluorescent intensity compared to spermatocytes recovered from MLN 8237 exposure for 1-h, although not from untreated control spermatocytes (Fig. 4, panel 4). This observation is surprising given the critical role phosphorylated TACC3 plays in regulating assembly of γ-TuRC for controlling microtubule nucleation ^rev,^^28^. Additionally, spindle microtubules in spermatocytes treated with MLN 8237 were largely disassembled despite this increase in spindle pole γ-tubulin intensity. The opposite occurred upon MLN 8237 washout- partial spindle microtubule recovery with a significant decrease in spindle pole γ-tubulin intensity (Fig. 4, panel 1). In siRNA-TACC3 depleted human cells, centrosomal γ-tubulin detection was either not impacted or significantly reduced at spindle pole^25,26,28,54^. More recently, human mitotic cells expressing TACC3 shRNA-resistant mutant proteins engineered to be incapable of binding ch-TOG at the spindle poles, thus uncoupling TACC3 from cKAP5/chTOG activity at the centrosomes, also demonstrated an increase in spindle pole γ-tubulin because of elevated spindle pole TACC3 phosphorylation increasing γ-TuRC protein recruitment to the poles^27^. Here, it is not clear why spindle pole γ-tubulin intensity increased after MLN 8237 disruption of TACC3. Perhaps experimental variability is a factor, given our spermatocyte cells are treated for an hour with MLN 8237 inhibitor as opposed to transfection protocols requiring hours-to-days to initiate impacts on spindle pole TACC3. Alternatively, perhaps other members of the TACC family of proteins (i.e. TACC1 or TACC2) could be active and compensating for the spindle pole TACC3 disruption in spermatocytes^55–57^.

Spermatocyte spindle pole TACC3 disruption by MLN 8237 also showed slightly reduced spindle pole cKAP5/chTOG with a significant increase of cKAP5/chTOG fluorescent intensity detected within the central spindle apparatus, but without specific binding to assembled spindle microtubules (Fig. 5). In human cells, siRNA-TACC3 spindle pole disruption did not impact centrosomal chTOG but did significantly reduced spindle microtubule ch-TOG binding^29^. More recently, uncoupling spindle pole TACC3 from ch-TOG significantly reduce spindle pole ch-TOG detection with a concomitant increase in TACC3-free ch-TOG binding to intra-spindle microtubules^27^. Taken together, the TACC3: cKAP5/chTOG dyad are critical regulators in male meiotic spindle pole organization with direct impacts on spindle microtubule organization, density, spindle pole length, and meiotic chromosomal alignment, observations unlike reports in human mitotic cells^29,58,59^ but perhaps like reports in mouse meiotic spindles^45–47^.

The TACC3 LISD is unique to many meiotic oocytes in mammals^47^. In GFP CETN2-expressing females, TACC3 is found in late GV-stage oocytes on expanding GV-residing and cytoplasmic MTOCs (Suppl Fig. S3), stages that often assemble short astral microtubules just before meiosis onset^15^. After meiosis resumption, TACC3 accumulates around cytoplasmic MTOC’s but is lost on spindle pole MTOCs and GFP CETN2-expressing foci^46^. TACC3 detection is significant in both the LISD and anastral meiotic spindle and kinetochore microtubules (Suppl Fig. S3 G–I)^45–47^. TACC3 disruption by siRNA, the AURKA inhibitor MLN 8237, or Trim Away depletion of endogenous TACC3 protein produced atypical meiotic spindle phenotypes with misaligned chromosomes and delayed or arrested meiotic progression by interfering with LISD integrity and TACC3 on spindle microtubules^41–47,60^. Similar observations on aberrant metaphase I spindles phenotypes were reported when the LISD could not assemble in oocytes from an Aurora A knockout mouse mutant or a transgenic mouse engineered to lack spindle pole MTOCs and associated phosphorylated AURKA^45,46^. Interestingly, a study using phosphorylated TACC3 (p-TACC3)-specific antibody showed restricted spindle pole MTOC association only and siRNA TACC3 depletion removed this MTOC-associate p-TACC3 from the spindle poles producing abnormal lengthened bipolar spindles with misaligned chromosomes^60^.

Like TACC3 detection in mouse oocytes, cKAP5/chTOG is absent from spindle pole MTOCs and associated GFP CETN2-expressing foci but does immunolabel meiotic spindle and kinetochore microtubules (Suppl Fig. S4)^43^. But, unlike TACC3, cKAP5/chTOG maintains cytoplasmic MTOC colocalization after meiosis resumption. While unknown, perhaps TACC3 and cKAP5/chTOG association with mouse oocyte cytoplasmic MTOC’s may participate in directing the first meiotic spindle positioning to the cortex^61^.

In mouse oocytes, TACC3 is concentrated in a liquid-like condensate (LISD) disassembled by exposure to the disrupting agent 1,6-hexanediol^51,52^. Mouse oocyte spindles treated with 1,6-hexanediol depleted a majority of TACC3 LISD but remained bipolar with TACC3 detected on spindle pole microtubules^47^. In spermatocytes, application of 3.5% 1,6-hexanediol for 30 min removed all spindle pole TACC3 detection, disrupting normal bipolar spindle organization, spindle pole centriole GFP CETN2 expression and/or locality, and chromosome alignment (Fig. 6). Unlike MLN 8237 AURKA inhibitor treatment, spermatocytes significantly recovered centrosomal TACC3 after hexanediol washout, with good spindle bipolarity, tightly focused spindle pole microtubules but overall reduced microtubule density and unaligned chromosomes (Fig. 6, panel 1). Analysis after recovery from 1,6-hexanediol showed significantly higher spindle pole microtubule intensity measurements when TACC3 could reform at the centrosomes as opposed to recovered spermatocytes from MLN 8237 treatment that did not restore spindle pole TACC3, although both treatments were significantly reduced in spindle pole microtubules compared to control spermatocytes (Fig. 6, panel 3). Finally, we investigated male meiotic spindle assembly in cold depolymerization-rescue experiments in the presence of 1,6-hexanediol that depletes spindle pole TACC3 (Fig. 7). We observed robust microtubule aster assembly from the sperm centrosomes at both spindle poles, despite severely reduced centrosomal TACC3 detection but abundant cytoplasmic or cortical TACC3 aggregates. Yet, we observed incomplete bipolar spindle assembly and no meiotic chromosome realignment. Taken together, these findings support observations that spindle pole TACC3 in spermatocytes does not influence initial microtubule assembly from male spermatocyte centrosomes but plays a critical role in organizing spindle poles to support and stabilize male meiotic bipolar spindles.

Spermatocyte spindle pole TACC3 and cKAP5/chTOG appear independent of spindle microtubule dynamics based on analysis of spermatocytes treated with the microtubule depolymerization drug nocodazole or the microtubule stabilizing drug paclitaxel. Neither drug treatment significantly impacted centrosomal TACC3 or cKAP5/chTOG detection in spermatocytes (Suppl Fig. S5), supporting observations in human mitotic cells^29,62^. But, in mouse female oocytes, nocodazole exposure removed meiotic spindle microtubules to expose TACC3 protein, but not cKAP5/chTOG, in the unique LISD^43,47^. Taxol enhanced meiotic oocyte spindles, including astral microtubules at the poles, were all immunolabeled with cKAP5/chTOG^43^. We conclude that microtubule dynamics probably are not significant in maintaining spindle pole TACC3 or cKAP5/chTOG localization in male meiosis, as reported in mitotic cells, but distinctly different from mouse female meiotic oocytes.

Figure 8 summaries our preliminary findings for TACC3 and cKAP5/chTOG in male meiotic spindles. We provide evidence for similarities and differences on spindle assembly and maintenance mechanisms for preserving accurate chromosome segregation in male and female meiosis versus somatic mitotic cells. TACC3 and cKAP5/chTOG are remarkable divergence in spindle localization with overlapping but also unique functions in supporting spindle integrity. With nearly 200 essential microtubule associated proteins identified mediating spindle assembly and disassembly activities, the foundations for understanding the molecular underpinnings of meiotic and mitotic spindle assembly in directing proper chromosome segregation lay ahead. Understanding their commonalities and distinctions will help avoid chromosomal separation errors that lead to infertility, genetic and developmental defects, as well as cancers.

## Methods

### Mouse husbandry, handling, and Institutional oversight

All animal procedures were approved by the Institutional Animal Care and Use (IACUCs) Committees at the University of Pittsburgh and Magee-Womens Research Institute (protocol #22010530) in compliance with the National Institute of Health’s Office of Laboratory Animal Welfare *Guide for the Care and Use of Laboratory Animals* and the ARRIVE guidelines. CB6-Tg (CAG-EGFP/CETN2)3-4Jgg/J mice (Stock number: 00823445) were obtained from the Jackson Laboratory (Bar Harbor, ME) as juveniles, bred, and analyzed as described previously^15,17^. All mice were housed in an Association for Assessment and Accreditation of Laboratory Animal Care (AAALAC)-accredited mouse facility and tissues collected after humanely euthanizing mice by carbon dioxide (CO_2_) asphyxiation (55–60 cubic feet per hour flow rate; 2–4 min) followed by cervical dislocation using approved methods by the American Veterinary Medical Association (AVMA) and our University-approved IACUC protocols. Tissues were harvested within 5 min post-euthanization. We investigated isolated spermatogenic cells from 54 testes from 27 male mice and produced 983 spermatogenic cells for analysis.

*GFP expression determination by PCR.* Genomic DNA was isolated for GFP detection in GFP CETN2-expressing mice, using tail tip tissues (< 5 mm) and PCR with MyTaq Extract-PCR Kit (Bioline, Taunton, MA) as previously described^17^.

### Spermatogenic cell isolation from seminiferous tubules

Seminiferous tubules (SfTu) from GFP CETN2-expressing male testes were collected after euthanasia as previously described^17^ and placed in modified Enriched Krebs–Ringer Bicarbonate medium (mEKRB) consisting of 120 mM NaCl, 4.8 mM KCl, 4 mM NaHCO_3_,1.2 mM KH_2_PO_4_, 1.2 mM MgSO_4_·7H_2_O, 1.3 mM CaCl_2_·2H_2_O, 10 mM HEPES, 11.1 mM dextrose, 1000U/ml and 1000 µg/ml Pen/Strep, 1:50 of 50 × essential amino acids, and 1:100 of a 100× nonessential amino acids (components from Thermo Fisher, Waltham, MA, or Sigma-Aldrich, St. Louis, MO)^63^. After mechanical removal of the tunica albuginea and fat pad material in mEKRB, SfTu were manually cut into ~ 20-mm-long pieces with sterile scissors. Cut SfTu were transferred to a 50-ml Falcon tube and allowed to settle for 5 min in mEKRB at 34 °C. After decanting supernatant, SfTu were suspended in 5 ml of 1 mg/ml collagenase IV in mEKRB and agitated at 37 °C for 3–5 min, washed twice in mEKRB by gravity sedimentation, and then incubated in 5 ml of 0.05% trypsin:0.53 mM EDTA (Thermo Fisher) for 25 min with rocking agitation in a 37 °C/5% CO_2_ incubator. After SfTu digestion, an equal volume of mEKRB with 4 mg/ml BSA was added to neutralize the trypsin:EDTA solution and incubated at 34 °C for 10 min. The cell suspension was then passed through a 100-micron filter (Fisher Scientific, Pittsburgh, PA) into a 50-ml Falcon tube, centrifuged at 125×*g* for 3 min to collect cells, and the pelleted cells were resuspended in mEKRB (no BSA) for further treatment (see below) or fixation on poly-lysine-coated coverslips.

### Seminiferous tubule drug exposure and spermatogenic cell isolation post-fixation

GFP CETN2-expressing male SfTu were collected and prepared as above. After manual dissection of tubules into 20-mm-long pieces and pretreatment with 5 ml of 1 mg/ml collagenase IV in mEKRB, tubules were washed twice in mEKRB by gravity sedimentation. SfTu were separated into 50-ml conical tubes as control (no treatment), drug-exposure, or drug-exposure-with-rescue groups. All washes to remove drug exposure were performed by gravity settling at 34 °C. Following treatment, SfTu were fixed in an equal volume of 4% paraformaldehyde (pFA) in mEKRB for 30 min at 34 °C. Tubules were then further cut into 1.5- to 3-mm pieces with scissors in 100 µl of mEKRB on a 2-well chamber slide (Nunc; Thermo Fisher). A 25-mm round plastic coverslip (Thermanox; Ted Pella, Redding, CA) was cut to fit the well, and pressure was applied to squash the fixed SfTu and release fixed cells as described^64^. Released cells were harvested from the wells using a plastic transfer pipet with tip cut to prevent damage, and cells were placed in the center of a 22-mm^2^ poly-lysine-coated slide (2 mg/ml; Sigma-Aldrich). After 5 min, attached cells were washed in PBS + 0.25% Triton X-100 detergent (PBS-TX) to remove unbound cells before further processing for immunocytochemistry staining as below.

### Primary mouse embryonic fibroblast cell culture, Lentiviral transduction with CETN2-GFP and fixation

Commercially available primary mouse embryonic fibroblasts isolated from CF-1 mice (Millipore; catalog # PMEF-CF) were thawed and propagated using protocols according to the manufacturer. Cells were grown in the Feeder Cell Medium (DMEM; 10% Fetal Bovine Serum; 1% NEAA, 100x; 1% penicillin–streptomycin, 100×; 1% L-glutamine solution, 100x). Media was exchanged with fresh media every other day, and cells were dissociated enzymatically with TrypLE (Thermo Fisher) every 3–5 days.

For transduction with lentiviral CETN2-GFP (a generous gift from Dr. Jeffrey Salisbury; Mayo Clinic, Rochester, MN), viral particles were produced using the ViraPower Lentiviral Packaging Mix (Thermo Fisher) according to the manufacturer’s recommendations. CF-1 primary mouse embryonic fibroblast cell lines were transduced after plating cells into a T-25 cell culture-treated flask at 37 °C overnight with viral particles and 6 mg/mL of polybrene (Millipore). After 24 h, transduced cells were washed once with PBS, and transduction medium was replaced with Feeder Cell Complete cell culture medium. Confirmation of centriole and centrosome expression was confirmed by indirect immunofluorescence on a Nikon inverted microscope equipped with appropriate filters for visualizing GFP.

Fixation of GFP CETN2 CF-1 primary fibroblasts was accomplished in 1% paraformaldehyde in DMEM without protein for 40 min at 37 °C after overnight seeding on sterile 22-mm^2^ coverslips. After PBS washes, coverslips were permeabilized, blocked, and stained as described below.

### Mature germinal vesicle (GV)-stage oocyte collection and zona-free in vitro fusion with spermatogenic cells, using Sendai extract

Mature GV-stage oocytes were collected from GFP CETN2-expressing CB6F1 ovaries in EmbryoMax M2 (EMD Millipore, Billerica, MA) in the presence of 100 µg/ml dibutyryl adenosine 3′,5′-cyclic monophosphate (dbcAMP; Sigma-Aldrich) to prevent in vitro maturation as previously described^15^. Briefly, sterile excised ovaries were minced mechanically using sterile needles to collect GVs. Cumulus cells were removed with a 75-µm tip (Stripper pipet; Origio Mid-Atlantic Diagnostics, Trumbull, CT). Mature GV oocytes ≥ 65 µm in diameter were segregated from the population of GV’s after determining diameters using Elements software (Nikon USA, Melville, NY) and after image capture on a Nikon Digital Sight camera (DS Fi1). GV oocyte maturation was initiated after 3× washes in M-2 and placing mature GVs in advanced KSOM (Sigma-Aldrich) at 37 °C in a humified 5% CO_2_ incubator until fixation.

To accomplish zona-free GV oocyte in vitro fusion with isolated spermatogenic cells, we utilized an inactivated Sendai virus cell fusion kit (HVJ Envelope: HVJ-E; Cosmo Bio, Tokyo, Japan) as previously described^17^. Briefly, zona-free GV oocytes were prepared by a 35- to 45-s treatment with warm EmbryoMax acidic Tyrode’s culture medium (EMD Millipore), washed 3 × in M-2, and then placed in individual 15-μl droplets of ice-cold 1× fusion buffer containing a 1:25 dilution of Sendai HVJ-E extract in a sterile 35-mm petri dish coated with parafilm to prevent zona-free oocyte adhesion. Isolated GFP-CETN2-expressing spermatocytes were laid over the zona-free oocytes in Sendai extract and pipetted repeatedly to adhere spermatocytes to the GV oocytes. Fusion of the spermatocyte with zona-free GV oocyte couplet was accomplished by gently flooding with 500-μl of warm 1× fusion buffer at 37 °C for 15 min. After fusion incubation, oocytes were transferred individually to 15-μl droplets of advanced KSOM medium under mineral oil until fixation at 2–4 h post-fusion and processed for immunocytochemistry analysis as described below.

### Cytoskeletal, Aurora A kinase inhibitor, cold and hexanediol treatment

10-µM stock solutions of nocodazole and paclitaxel (Sigma-Aldrich), along with 5 mM stock of MLN 8237 Aurora A Kinase inhibitor (Cayman Chemical, Ann Arbor, MI) were prepared in DMSO and stored at − 80 °C. All drug stocks were diluted to final concentrations in mEKRB. Rescue experiments from inhibitors for isolated spermatogenic cells were performed by centrifuging cells at 125×*g* for 3 min, discarding supernatant (inhibitor), and adding back 2 ml of inhibitor-free mEKRB to cell pellets for 15–60 min recovery at 34 °C. Cold treatment was performed in ice-cold water at 4 °C in mEKRB for 15 min, with rescue experiments performed by a 15-min recovery at 34 °C. Hexanediol (Sigma-Aldrich) was suspended in mEKRB at a final concentration of 3.5% just before use^47^.

### Immunocytochemistry

Isolated spermatogenic cells attached to poly-lysine-coated coverslips were fixed in 0.5% pFA (Electron Microscopy Services, Hatfeld, PA) for 30-min in a 37 °C Thermolyne incubator. Fixed GFP CETN2 CF-1 cells, mouse oocytes, and spermatogenic cells (either isolated cells or released from fixed SfTu) were first permeabilized in 2% PBS-TX for 30 min at room temperature before blocking in BlockAid (Thermo Fisher) for 40 min at room temperature. Primary antibody details, including RRID validation numbers, are presented in Supplemental Fig. S1 and include rabbit monoclonal TACC3 (Abcam, Boston, MA; ab134154), mouse monoclonal TACC3 (Abnova, Taipei, Taiwan; H00010460-M02), rabbit cKAP5/ch-TOG (Thermo Fisher; PA5-59150), rat YL12 tyrosinated α-tubulin (Novus Biologicals, Centennial, CO; NB 600–506) or YOL 1/34 (Millipore; NB 600-506), mouse 611B-1 anti-acetylated a-tubulin (Sigma-Aldrich; MABT868), mouse PCBD30 pericentrin (BD Transduction Laboratories; Franklin Lake, NJ; 611814), and mouse Tu30 anti γ-tubulin (Abcam; ab27074) or GTU88 anti γ-tubulin (Sigma-Aldrich; T5326), all diluted to final concentrations (Suppl Fig S1) in sterile PBS and applied overnight at 4 °C. Primary antibodies were detected with appropriate fluorescently tagged secondary antibodies (Thermo Fisher) at room temperature for 2 h in the dark after PBS rinses. DNA (final concentration of 10 µg/ml, each) was labeled with a combined DNA stain of Hoechst 33342 and DAPI from a 1 mg/ml stock solution for 10 min at room temperature before mounting in Vectashield antifade mounting medium (Thermo Fisher) and sealing with nail varnish.

### Equipment, imaging, analysis, and settings

Imaging of fixed slides was accomplished as described^17^. Briefly, images were collected with a Nikon A1 four-laser line confocal microscope equipped with Elements A1 Plus compact GUI acquisition software (version 4.20; Nikon USA) at 1024 × 1024 size at ¼ frame per second, using a pinhole size of 79.2 mm and a z-depth of 0.25 mm through the entire spermatogenic cell, with a differential interference contrast (DIC) Plan Fluor ×100 (1.4 NA) objective. We collected 5- × 12-bit depth images (nd2 files), using the same laser photomultiplier tube settings for each channel across specimens (5% laser power, except UV, for DNA imaging, at 10.24%) to facilitate comparison between meiotic spermatogenic control versus drug-treated cells. Fluorescent intensity ratios, surface intensity plots, area, or volume measurements were performed on binarized images, using the threshold tool and region-of-interest statistical menu in the Elements software, and downloaded to Microsoft Excel for statistical analysis. For image panel presentation, generated confocal nd2 files were first denoised in the A1 software before performing a subtracted background image, collected from outside of the spermatogenic cells. All channels were then subjected to the deconvolution software module in Elements (Landweber algorithm) using the point-scan confocal command and same filter (noisy) at twenty iterations for all images. Final panels from deconvolved images were prepared in Photoshop (Adobe Systems, San Jose, CA).

### Statistics

Means ± standard deviations were determined using calculator.net (Maple Tech International, LLC; The Woodlands, TX). We used Excel (Microsoft; Redmond, WA) to prepare graphs and box plots, which show median (horizontal lines), 25th and 75th percentiles (small boxes), and min and max (whiskers). Statistical significance was determined by Student’s T-test (GIGA calculator; Web Focus, LLC; Sofia, Bulgaria). Significance was determined at *p* < 0.05. Graphical analyses shown are indicative of average values ± standard deviation. For most experiments, more than three trials were performed, and data are representative of all trials.

### Supplementary Information

Supplementary Figures.

## Data Availability

Datasets generated and/or analyzed during this study are available from the corresponding author on reasonable request as set forth in the guidelines of this journal.
