# Supplementary material for: Male meiotic spindle poles are stabilized by TACC3 and cKAP5/chTOG differently from female meiotic or somatic mitotic spindles in mice
Source: Sci Rep. 2024 Feb 27;14:4808. doi: 10.1038/s41598-024-55376-z (PMC10899211; doi:10.1038/s41598-024-55376-z)
Supplement: Supplementary file 1 — Supplementary Figures. [file 41598_2024_55376_MOESM1_ESM.pdf]

SUPPLEMENTAL MATERIAL.

Male Meiotic Spindle Poles are Stabilized by TACC3 and cKAP5/chTOG Differently from Female Meiotic or Somatic Mitotic Spindles in Mice.

by

Calvin Simerly, Emily Robertson, Caleb Harrison, Sydney Ward, Charlize George, Jasmine Deleon, Carrie Hartnett, and Gerald Schatten[1]

Pittsburgh Development Center of Magee-Womens Research Institute, Departments of Cell Biology, Ob-Gyn-Repro Sci, and Bioengineering, University of Pittsburgh Medical Center, 204 Craft Avenue, Pittsburgh, PA 15213

[1] corresponding author; gschatten@pdc.magee.edu

Supplemental Fig. 1S: Antibodies Utilized in Mouse Meiotic Spermatogenic Cells

Antibody	Name	Host	Source	Cat. Number	IF Dilution	Localization/ Function	RRID number
**TACC3**	Transforming Acidic Coiled-Coil Containing Protein 3	Rabbit IgG (monoclonal)	Abcam	ab134154	1:100	Spindle MTs; centrososomes/MT nucleation and stability	No RRID number, validated using knockout cell line. RNAi validation (Furey, 2021)[1]
**TACC3**	Transforming Acidic Coiled-Coil Containing Protein 3	Mouse IgG (monoclonal)	Abnova	H00010460 -M02	1:100	Spindle MTs; centrososomes/MT nucleation and stability	AB_566223
**cKAP5/ chTOG**	Cytoskeletal Associated Protein 5/colonic and hepatic tumor overexpressed gene	Rabbit IgG (polyclonal)	Themo Fisher Scientific	PA5-59150	1:200	Centrosomes/ MT binding protein	AB_2639841 RNAi validation (Furey, 2021)[1]
**YL12**	Tyrosinated α-tubulin	Rat IgG2a (monoclonal)	Novus	NB 600-506	1:200	MTs/ Stains tyrosinated post-translationally modified MTs	AB_343284 (validation status unknown)
**YOL 1/34**	anti- α -tubulin	Rat IgG (monoclonal)	Millipore	CBL270	1:200	MTs/ Stains a-tubulin	AB_93477 (validation status unknown)
**611B-1**	Anti-acetylated α-tubulin	Mouse IgG (monoclonal)	Sigma-Aldrich	MABT868	1:100	MTs/stains acetylated post-translationally modified MTs	AB_2819178
**PCBD30**	pericentrin	Mouse IgG (monoclonal)	BD Transduction Laboratories	611814	1:200	Pericentrosomal material (PCM)/regulatecentrosome function, cell cycle checkpoints, spindle formation, and cytokinesis	AB_399294
**Tu30**	γ-tubulin	Mouse IgG (monoclonal)	Abcam	ab27074	1:200	Pericentrosomal material/ nucleates MTs from centrosomes	AB_2211240 (validation status unknown)
**GTU-88**	γ -tubulin	Mouse IgG (monoclonal)	Sigma-Aldrich	T5326	1:200	Pericentrosomal material/ nucleates MTs from centrosomes	AB_532292 (validation status unknown)

[1] Furey C, Astar H, Walsh D. Human Cytomegalovirus Exploits TACC3 To Control Microtubule Dynamics and Late Stages of Infection. J Virol. 2021 Aug 25;95(18):e0082121. doi: 10.1128/JVI.00821-21. Epub 2021 Aug 25. PMID: 34191581; PMCID: PMC8387038

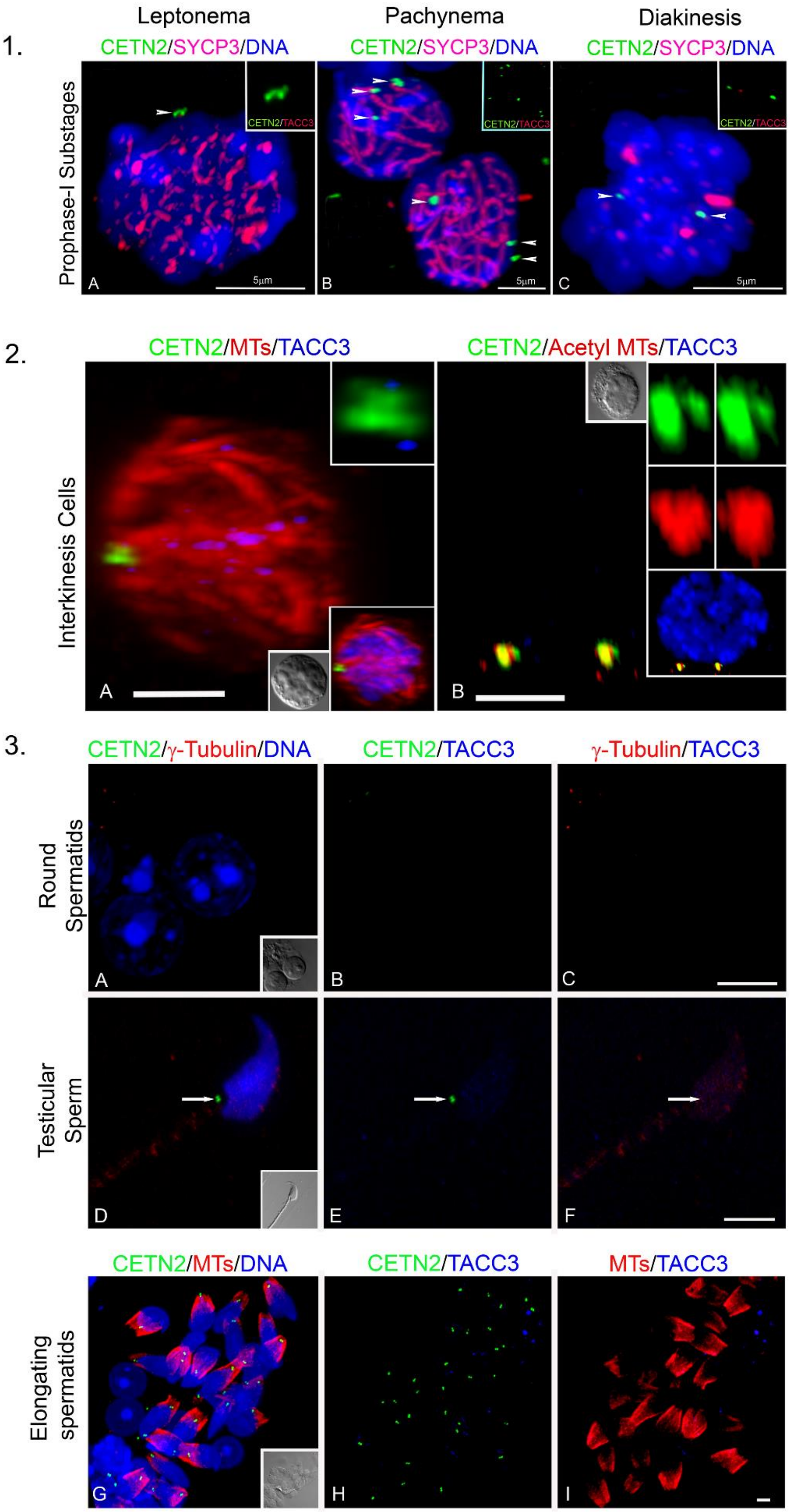

1.
Leptonema
CETN2/SYCP3/DNA
Pachynema
CETN2/SYCP3/DNA
Diakinesis
CETN2/SYCP3/DNA
Prophase-I Substages
CETN2/TACC3
A
5µm
B
5µm
C
5µm
2.
CETN2/MTs/TACC3
CETN2/Acetyl MTs/TACC3
Interkinesis Cells
A
B
3.
CETN2/γ-Tubulin/DNA
CETN2/TACC3
γ-Tubulin/TACC3
Round Spermatids
A
B
C
Testicular Sperm
D
E
F
CETN2/MTs/DNA
CETN2/TACC3
MTs/TACC3
Elongating spermatids
G
H
I

Supplemental Figure S2. TACC3 does not immunolabel male mouse prophase-I substages, interkinesis cells, post -meiotic spermatids or testicular sperm. *Panel 1*: Mouse spermatocyte prophase- I substages leptonema (A), pachynema (B) and diakinesis (C). A-C: GFP- expressing CETN2 centrioles (green), anti- SYCP3 (red), DNA (blue). Insets: TACC3 (red) and GFP CETN2 centrioles (green). TACC3 is absent in all prophase-I substages. *Panel 2*: Spermatocyte interkinesis stages, pre-centriole duplication (A: green) and post centriole replication (B: green). A: TACC3 (blue) does not immunolabel microtubules (red) or centrioles (green). B: acetylated α-tubulin (red) co-immunolabels duplicated centrioles (green), but without TACC3 (blue). A, B: upper insets: GFP CETN2 (green) and TACC3 (blue); lower insets: GFP CETN2 (green), microtubules (red), and DNA (blue). Lower left, upper right insets: DIC image. B: middle insets: acetylated α-tubulin (red) and TACC3 (blue). *Panel 3*: Post-meiotic spermatocytes. A-C: Round spermatids with silenced GFP CETN2 (A-B: green), no γ-tubulin (A-C: red) or TACC3 (B-C: blue). D-F: Testicular sperm with GFP CETN2 centrioles (D-E: green, arrows) but no γ-tubulin (D-F; red) or TACC3 (E-F: blue). G-I: Elongating spermatids with GFP CETN2 centrioles (G-H, green), manchette microtubules (G, I: red) but no TACC3 (H-I: blue). A, D, G: blue, DNA; insets, DIC image. *Panel 1*: GFP CETN2-expressing prophase spermatocytes (green: A, B, C, insets) immunolabeled with SYPC3 (red: A, B, C), TACC3 (red, insets: A, B, C) and DNA stain (blue: A, B, C). *Panel 2*: GFP CETN2-expessing spermatocytes (green: A, B, upper insets) immunolabeled with YL1/2 microtubules (red: A, lower inset) or acetylated α-tubulin (red: B, middle inset), TACC3 (blue: A, B, lower insets) and DNA stain (A, B: lower insets). DIC: differential interference contrast image. *Panel 3*: GFP CETN2-expressing post-meiotic spermatocytes (green: A, B, D, E, G, H) immunolabeled for γ–tubulin (red: A, C, D, F) or YL1/2 MTs (red: G, I), TACC3 (blue: B, C, E, F, H, I) and DNA stain (blue: A, D, G). All scale bars= 5 µm.

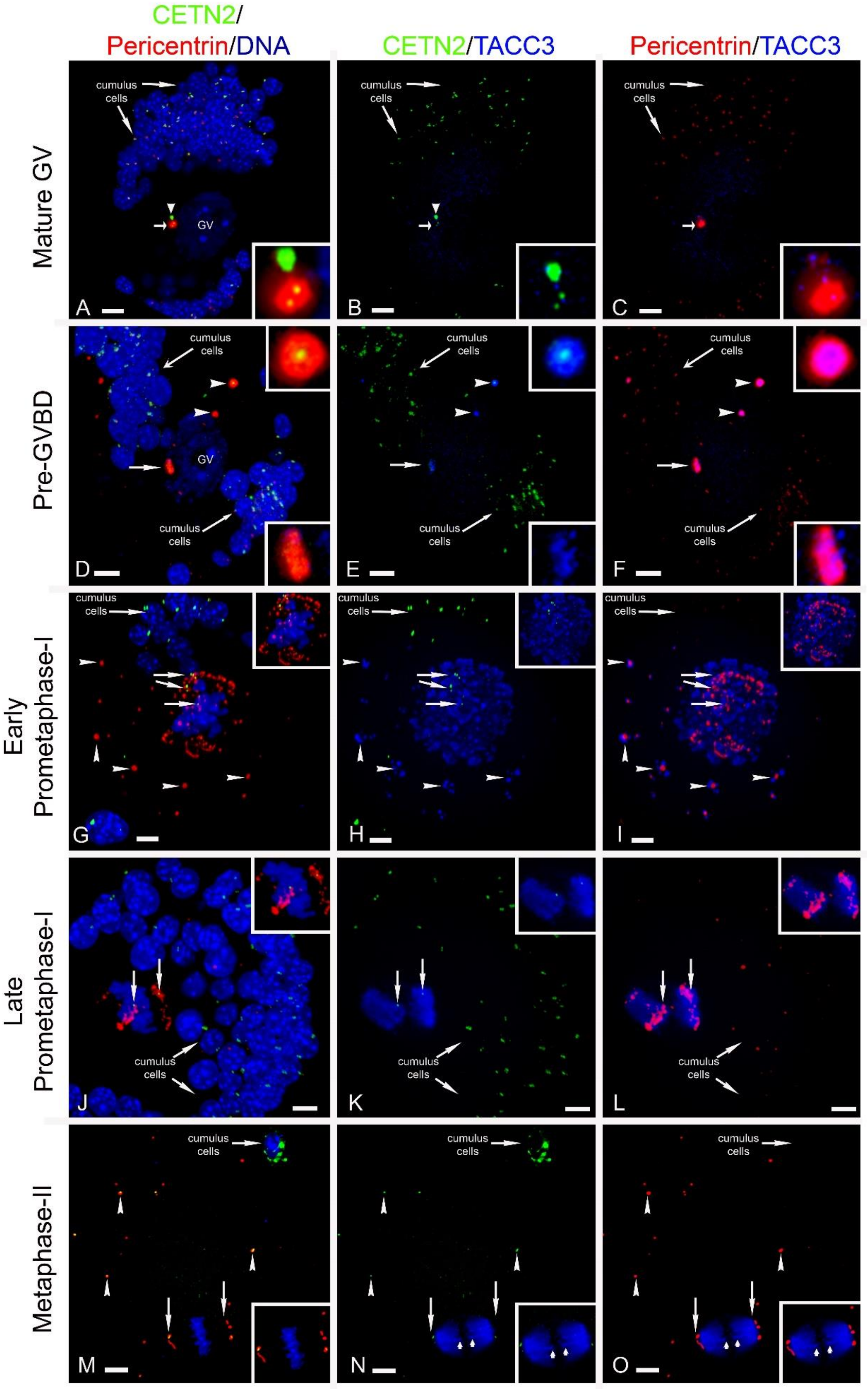

CETN2/
Pericentrin/DNA
CETN2/TACC3
Pericentrin/TACC3
Mature GV
Pre-GVBD
Early
Prometaphase-I
Late
Prometaphase-I
Metaphase-II
cumulus
cells
GV
A
B
C
D
E
F
G
H
I
J
K
L
M
N
O

Supplemental Figure S3. Mouse oocyte TACC3 initially localizes to the pre-germinal vesicle breakdown (GVBD) expanding MTOC's and then assembles at the spindle LISD and cytoplasmic MTOCs after meiosis resumption but does not specifically immunolabel maternal GFP-CETN2-expressing foci, spindle pole-residing MTOCs or somatic cumulus cell centrosomes. A-C: A GV-residing MTOC with pericentrin (A, C: red, short arrows), GFP-CETN2 foci (A-B: green; insets, details) but no TACC3 (B, C: blue; inset, details). A: blue, DNA. Cumulus cells (A-B: centrioles, green, long arrows) with centrosomal pericentrin (A, C: red) are without TACC3 (B, C: blue). A, B: green, arrowhead: GFP aggregate. D-F: Pre-GVBD oocyte (D: blue, DNA) with TACC3 GV-residing MTOC and cytoplasmic MTOC's (E, F: blue, short arrow, arrowheads; insets, details). MTOC pericentrin (D, F: red, short arrow, arrowhead; inset, details); GFP CETN2-expressing doublets (D, E: green, upper arrowhead); long arrows: GFP CETN2-expressing cumulus cell centrioles (D, E: green), centrosomal pericentrin (D, F: red) without TACC3 (E, F: blue). G-I: Early prometaphase-I oocyte (G: blue, DNA), fragmenting spindle MTOCs at opposing spindle poles (G, I: red), and GFP CETN2 foci at one pole (G, H: green, short arrows; insets, details). TACC3 LISD assembles beyond the spindle pole MTOC's (H, I: blue; insets, details). Cytoplasmic MTOCs (G, I: red, arrowheads) co-label or are encircled with TACC3 (H, I: blue, arrowheads) but not cumulus cells (G, H: centrioles: green, long arrows) with pericentrin (G, I: red). J-L: late prometaphase-I oocyte (J: blue, DNA), pericentrin spindle pole MTOC's (J, L: red; insets, details), and TACC3 LISD encompassing the spindle microtubules (K, L: blue; insets, details). Cumulus cells (J, K: centrioles; green, arrows; J, L: pericentrin: red) still lack TACC3 (K, L: blue). *M-O*: metaphase-II arrested oocyte (M: blue, DNA) with spindle pole pericentrin MTOC's (M: red), GFP CETN2-expressing foci (M: green; long arrows; inset, details), and spindle kinetochore microtubules TACC3 (N, O: blue; short arrows; inset, details). Arrowheads: cytoplasmic asters (M, O: pericentrin, red) with GFP CETN2 foci (M: green), but weak TACC3 (N, O: blue). Upper arrow: a degenerating cumulus cell (M, N: centrioles; green; M, O: pericentrin, red) lacking TACC3 (N, O: blue). Bars=10µm.

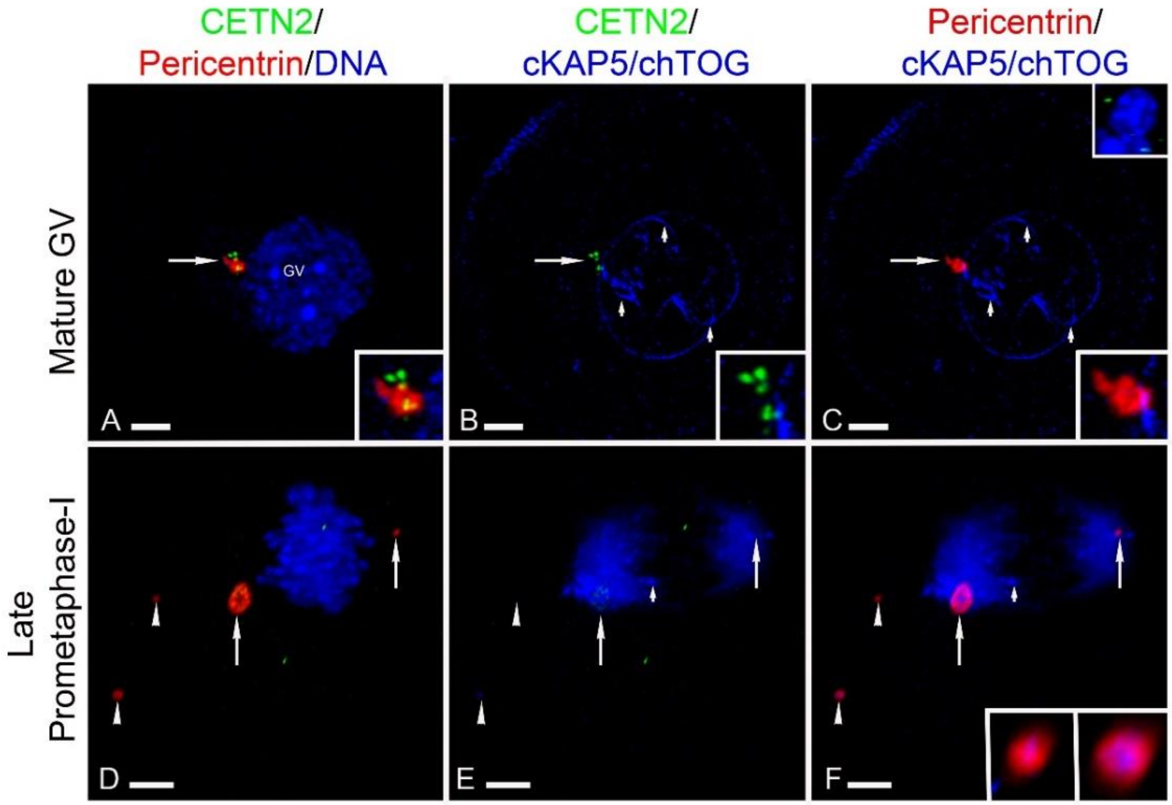

Supplemental Figure S4. **cKAP5/chTOG immunolabels the mouse oocyte GV nucleus, strongly associates with the spindle LISD after meiosis resumption, but not spindle pole MTOC's nor cumulus cell centrosomes***.* <u>A-C</u>: arrested GV oocyte (A; blue, DNA) with GFP CETN2-expressing foci (A, B: green) within the pericentrin-tagged GV-residing MTOC (A, C: red, arrows). cKAP5/chTOG detects the GV nucleus and nuclear envelop invagination sites (B, C: blue; small arrows), not the MTOC (B, C: blue, arrow). GFP-CETN2-expressing cumulus cells (C: upper inset, green; blue, DNA) do not immunostain with cKAP5/chTOG (C: upper inset; red). <u>D-F</u>: late prometaphase-I oocyte (D: blue, DNA) with pericentrin spindle pole MTOCs (D, F: red, long arrows) but no GFP CETN2-expressing foci (D, E: green). cKAP5/chTOG (F: blue) localizes to the spindle LISD including kinetochore microtubules (E, F: blue; small arrows) but not the spindle pole pericentrin-tagged MTOCs (F: red; long arrow). Cytoplasmic MTOCs (D, F: red, pericentrin; arrowheads) weakly immunolabel with cKAP5/chTOG (F: blue; arrowheads; insets, details). All images are GFP CETN2-expressing mouse oocytes (green: A, B, D, E) immunolabeled for pericentrin (red: A, C, D, F), cKAP5/chTOG (blue: B, C, E, F) and DNA stain (blue: A, D). Scale bars= 10 µm.

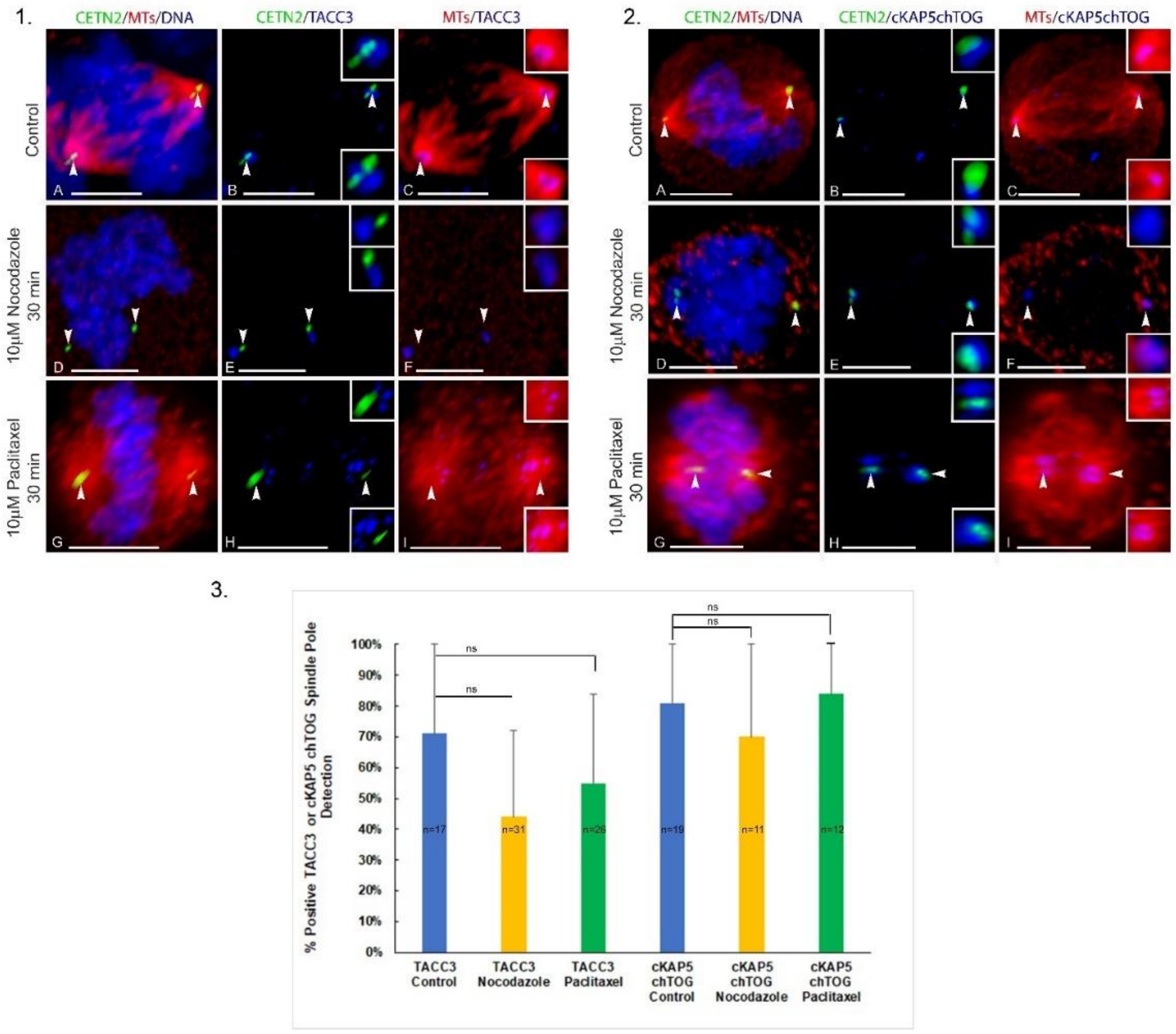

Supplemental Figure S5. The microtubule inhibitors nocodazole and paclitaxel do not significantly displace meiotic spindle pole TACC3 or cKAP5/chTOG localization in GFP CETN2-expressing meiotic spermatocytes despite interfering with normal spindle microtubule organization. *Panel 1*: TACC3 (B, C, E, F, H, I; blue) is found at the spindle pole GFP CETN2-expressing centrioles (B, E, H: green; arrowheads) after 30 min exposure to either the microtubule disassembly inhibitor 10µM nocodazole (D-F) or the microtubule stabilizing compound 10µM paclitaxel (G- I). TACC3 (E, F, H, I; blue) persists at spindle poles centrioles (E, F, H, I; green) after microtubule disassembly (D, F) in 10μM nocodazole (D, F: red) or enhanced spindle microtubules after exposure to 10µM paclitaxel (G, I: red). A-C: a metaphase control GFP CETN2-expressing spermatocyte (A, B: green) with a bipolar spindle (A, C: red, microtubules), aligned chromosomes (A: blue), and spindle pole TACC3 (B, C: blue). Insets: details of TACC3 (blue) at the GFP CETN2 centrioles (B, E, H: green) or spindle microtubules

(C, F, I: red). Scale bars=5µm. *Panel 2*: cKAP5/chTOG spindle pole detection (B, C, H, C, F, I: blue) in mouse GFP CETN2-expressing spermatocytes (A, B, D, E, G, H: green, arrowheads) after 30 min exposure to 10µM nocodazole (D-F) or 10µM paclitaxel (G-I). cKAP5/chTOG (E, F, H, I) is strongly detected at the spindle pole centrioles after complete microtubule disassembly (D, F: red) or enhanced spindle microtubule assembly following paclitaxel exposure (G, I: red). A-C: a control metaphase GFP CETN2-expressing (A, B: green, arrowheads) spermatocyte with slightly misaligned chromosomes (A: blue), a bipolar spindle (C: red, microtubules) and spindle pole cKAP5/chTOG (B, C: blue). Insets: details of cKAP5/chTOG at the GFP CETN2-expressing centrioles (B, E, H: green, arrowheads) or spindle pole microtubules (C, F, I: red). Scale bars=5µm. *Panel 3*: Analysis of GFP CETN2-expressing spermatocytes exposure to 10µM nocodazole or 10µM paclitaxel for 30 minutes did not show significant reduction of spindle pole TACC3 or cKAP5/chTOG (TACC3: nocodazole vs control: $p < 0.0629$; paclitaxel vs control: $p < 0.1478$; cKAP5/chTOG: nocodazole vs control: $p < 0.4774$; paclitaxel vs control: $p < 0.2681$); ns= not significant.
